# Caspase-2 kills cells with extra centrosomes

**DOI:** 10.1126/sciadv.ado6607

**Published:** 2024-10-30

**Authors:** Dario Rizzotto, Vincenza Vigorito, Patricia Rieder, Filip Gallob, Gian Mario Moretta, Claudia Soratroi, Joel S. Riley, Florian Bellutti, Stefano Li Veli, Alessia Mattivi, Michael Lohmüller, Sebastian Herzog, Beat C. Bornhauser, Etienne D. Jacotot, Andreas Villunger, Luca L. Fava

**Affiliations:** ^1^ CeMM Research Center for Molecular Medicine of the Austrian Academy of Sciences, 1090 Vienna, Austria.; ^2^ Armenise-Harvard Laboratory of Cell Division, Department of Cellular, Computational and Integrative Biology-CIBIO, University of Trento, Trento, Italy.; ^3^ Institute for Developmental Immunology, Biocenter, Medical University Innsbruck, 6020 Innsbruck, Austria.; ^4^ Department of Oncology and Children’s Research Centre, University Children’s Hospital Zürich, 8032 Zürich, Switzerland.; ^5^ Inserm U1268, Medicinal Chemistry and Translational Research, Paris F-75006, France.; ^6^ Faculté de Pharmacie, UMR 8038 CiTCoM, Université Paris Cité, Paris F-75006, France.

## Abstract

Centrosomes are membrane-less organelles that orchestrate a wide array of biological functions by acting as microtubule organizing centers. Here, we report that caspase-2–driven apoptosis is elicited in blood cells failing cytokinesis and that extra centrosomes are necessary to trigger this cell death. Activation of caspase-2 depends on the PIDDosome multi-protein complex, and priming of PIDD1 at extra centrosomes is necessary for pathway activation. Accordingly, loss of its centrosomal adapter, ANKRD26, allows for cell survival and unrestricted polyploidization in response to cytokinesis failure. Mechanistically, cell death is initiated upstream of mitochondria via caspase-2–mediated processing of the BCL2 family protein BID, driving BAX/BAK-dependent mitochondrial outer membrane permeabilization (MOMP). Remarkably, BID-deficient cells enforce apoptosis by engaging p53-dependent proapoptotic transcriptional responses initiated by caspase-2. Consistently, BID and MDM2 act as shared caspase-2 substrates, with BID being kinetically favored. Our findings document that the centrosome limits its own unscheduled duplication by the induction of PIDDosome-driven mitochondrial apoptosis to avoid potentially pathogenic polyploidization events.

## Teaser

Extra centrosomes can kill blood cancer cells by caspase-2-mediated proteolysis of two alternative substrates, BID and MDM2.

## INTRODUCTION

Centrosomes are membrane-less organelles involved in multiple biological processes, including mitotic spindle pole organization, cell migration, immune-synapse formation, and ciliogenesis (*1*, *2*). Lack of centrosomes has been causally linked to defective neurodevelopment and neurological disorders caused by the death of neuronal progenitor cells (*3*), a condition due to mutation of structural components of the centrosome found in human microcephaly (*4*). Excess centrosomes instead can impair faithful microtubule-kinetochore attachments in pseudo-bipolar mitotic spindles or promote multi-polar mitoses, thereby increasing the risk of cells acquiring an aneuploid karyotype (*5*, *6*). Unsurprisingly, centrosome abnormalities are frequently found in cancer and forced centrosome overduplication by PLK4 overexpression, a key regulator of their biogenesis, has been reported to suffice to promote cancer formation in animal models (*7*). Whether extra centrosomes are cause or consequence of malignant transformation is still debated, as healthy cells respond with p53-induced cell cycle arrest to their accumulation (*8*). Consistently, loss of p53 facilitates proliferation of immortalized cells overexpressing regulators of centriole duplication (*9*). These observations firmly establish p53 as a barrier against aneuploidy and transformation resulting from extra centrosomes. Whether this barrier is imposed solely by p53-dependent cell cycle arrest or whether it involves additional effector mechanisms remains uncertain.

Caspases are a class of endopeptidases that are recognized arbiters of cell death and inflammation. Caspase-2 is the most evolutionarily conserved member of the caspase family and is activated in the PIDDosome multi-protein complex (*10*, *11*). Its overall role in apoptosis, however, has remained a matter of intense debate. Triggers reported to promote caspase-2 activation include protein aggregation upon heat shock, endoplasmic reticulum (ER) stress, chronic spindle assembly checkpoint (SAC) activation, DNA damage, and G_2_-M checkpoint override (*12*–*15*). However, studies using multiple cell types from animals lacking individual PIDDosome components, including PIDD1 and RAIDD, failed to provide support for a rate-limiting role of caspase-2 in cell death initiation in response to this broad array of triggers (*16*–*18*). Regardless of these discrepancies, caspase-2 has been assigned tumor suppressor potential in animal models of *MMTV/c-neu*–driven breast cancer, ataxia-telangiectasia mutated (ATM) loss, and MYC-driven lymphomagenesis (*19*–*21*). Of note, in the latter, it was suggested to act by deleting aneuploid cells, as mouse embryonic fibroblasts and MYC-driven B cell lymphomas lacking caspase-2 showed increased frequencies of abnormal karyotypes (*22*, *23*). Moreover, a large-scale clinical study documented reduced caspase-2 expression levels to correlate with increased aneuploidy tolerance in colorectal cancer patients lacking the WNT pathway modulator BCL2L9 (*24*). Consistent with the idea that caspase-2 can kill aneuploid cells, loss of caspase-2 in HCT116 colon cancer cells provided partial protection from cell death induced by the aneuploidy-inducing reagent reversine, an inhibitor of MPS1 kinase, crucial for SAC activity (*24*). However, reversine also induces cytokinesis failure in a substantial fraction of cells traversing mitosis in the absence of a functional SAC (*8*). Together, these observations suggest that caspase-2 has a role in limiting the survival or the proliferative potential of aneuploid cells. Yet, it still remains unclear whether the activating cue for caspase-2 resides in the aneuploidy itself or rather a feature preceding or arising concomitantly to aneuploidy, such as extra centrosomes. Moreover, caspase-2 effectors promoting cell death are poorly defined. The proapoptotic BCL2 family protein BID, a member of the BH3-only subgroup, has been reported as a caspase-2 substrate and implicated in subsequent cell death initiation. Yet, most triggers reported to engage caspase-2 for apoptosis induction, such as DNA damage or ER stress, do not seem to universally rely on BID being present (*25*, *26*). BID plays a well-established role in connecting the intrinsic mitochondrial and extrinsic apoptosis pathways in response to death receptor activation. For this function, it needs to be processed by caspase-8 into its active truncated form, tBID (*27*). However, a physiological trigger driving processing of BID in a strictly caspase-2–dependent manner remains to be defined.

p53 activation in response to centrosome accumulation requires the assembly of the PIDDosome, a multi-protein complex able to activate pro–caspase-2. While early studies placed the PIDDosome downstream of p53 in the context of DNA damage–induced apoptosis, with PIDD1 being a transcriptional p53 target (*11*, *28*), our recent findings document that the PIDDosome can also act upstream, promoting caspase-2–dependent proteolysis of MDM2, leading to p53 accumulation in cells with extra centrosomes (*8*). Of note, the ability of the PIDDosome to respond to extra centrosomes is tightly linked to the presence of extra mature parent centrioles. Mechanistically, physical association of PIDD1 with mature parent centrioles requires centriolar distal appendages and ANKRD26 as the connecting element (*29*, *30*). Similar to the loss of any of the PIDDosome components, PIDD1, RAIDD, or caspase-2, ANKRD26 deficiency abrogates MDM2 cleavage and p53 pathway activation. This response is engaged in cancerous and immortalized epithelial cell lines that accumulate centrosomes, as well as in primary hepatocytes that undergo scheduled cytokinesis failure during development or regeneration (*8*, *31*). Curiously, in tissue culture, cells carrying extra centrosomes lose them over time due to strong counterselection (*6*, *32*). Whether this phenomenon is solely due to impaired proliferation of cells that fail to normalize their centrosome number or active promotion of cell death downstream of centrosome accumulation remains uncertain.

Considering the purported role of caspase-2 in promoting apoptosis of aneuploid cells as opposed to promoting cell cycle arrest in cells accumulating centrosomes, it is presently unclear whether different cues lead to different outcomes upon caspase-2 activation, or rather cell type and context drive the fate switch once caspase-2 becomes activated by a common trigger. Here, we provide evidence that caspase-2 promotes apoptosis downstream of extra centrosomes by targeted proteolysis of its shared substrates, BID and MDM2. Our findings reconcile a vast set of seemingly contradictory reports in the field on the apoptotic contribution of caspase-2 and provide a rationale for the activity of a new class of antimitotic agents targeting the SAC and mitotic progression, such as Aurora kinase, MPS1 kinase, or CENP-E inhibitors.

## RESULTS

### Mitotic perturbation leads to variable outcomes depending on the cell type

Initially, we analyzed the response of different cell types with functional p53 status to different inhibitors of mitotic progression or cytokinesis, reported to promote p53 activation. These included the spindle poisons nocodazole and Taxol, which activate the SAC. We also used a range of inhibitors that promote cytokinesis failure, including dihydrochalasin B (DHCB), the Aurora kinase inhibitor ZM447439 (ZM), or a combination treatment of Taxol with reversine, an MPS1 kinase inhibitor. Reversine, overriding the SAC, forces cells to rapidly progress through mitosis irrespective of the presence of unattached kinetochores. We exposed human A549 lung cancer cells, the immortalized hTERT-RPE1 retinal pigmented epithelial (RPE1) cells and Nalm6 pre-B acute lymphoblastic leukemia (ALL), as well as murine BaF3 pro-B cells to the aforementioned treatments (fig. S1A). While nocodazole did not induce significant death in epithelial cells, Taxol did so quite potently in all the cell lines tested, epithelial and hematopoietic alike. However, we also noted that epithelial cell lines consistently entered cell cycle arrest after treatment with cytokinesis failure–inducing drugs, while hematopoietic cells readily committed to apoptosis (fig. S1B).

### An unbiased genetic screen identifies the PIDDosome as an activator of mitochondrial apoptosis in cells that fail cytokinesis

**Fig. 1.**
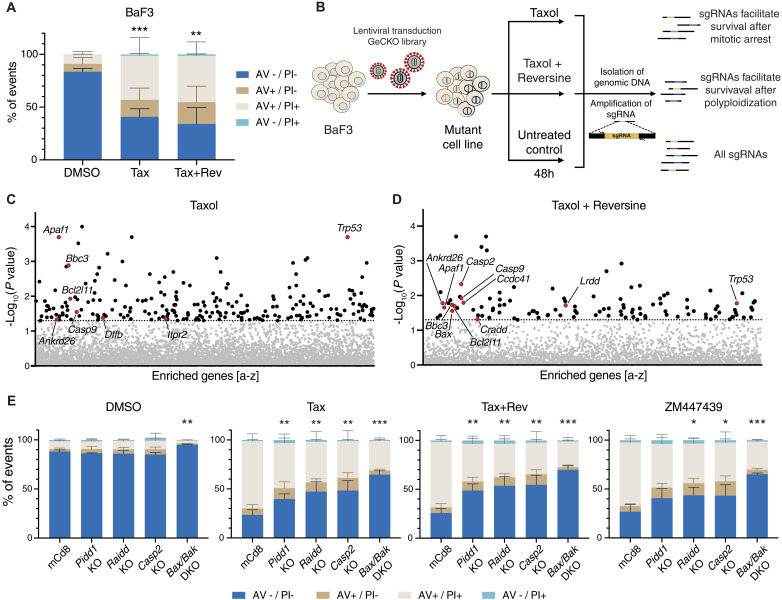
A CRISPR screen identifies genes involved in the induction of cell death after mitotic perturbation. (**A**) BaF3 cells were treated for 48 hours with 50 nM Taxol (Tax) alone or in combination with 500 nM reversine (Tax + Rev) before staining with annexin V (AV) and propidium iodide (PI) followed by flow cytometric analysis. Data are presented as means ± SD of the percentage of events in each staining condition. AV−/PI− = live cells; AV+/PI− = early apoptosis; AV+/PI+ and AV−/PI+ = late apoptosis. *N* = 3 independent biological replicates. Statistical significance calculated by unpaired *t* test on the percentage of live cells relative to the DMSO control. (**B**) Scheme of the CRISPR screen experimental setup. (**C**) Enriched sgRNAs found in surviving BaF3 cells after Taxol treatment (50 nM). Horizontal dashed line indicates the significance *P* value cutoff (0.05). See also fig. S2A. (**D**) Enriched sgRNAs in surviving BaF3 cells after Taxol + reversine treatment (50 nM + 500 nM). Horizontal dashed line indicates the significance *P* value cutoff (0.05). See also fig. S2B. (**E**) Annexin V/PI staining and flow cytometric analysis of control (mCd8), Pidd1 KO, Raidd KO, Casp2 KO, and Bak + Bax double KO (DKO) cells, 48 hours after treatment with Taxol (50 nM), Taxol + reversine (50 nM + 500 nM), ZM (2 μM), or DMSO. Data are presented as means ± SD of the percentage of events in each staining condition. *N* ≥ 3 independent biological replicates. Statistical significance calculated by unpaired *t* test on the percentage of live cells of each derivative clone relative to the mCd8 control for each drug treatment. See also fig. S2C. *P < 0.05; **P < 0.01; ***P < 0.001.

To identify regulators and effectors of cell death induced by SAC activation or cytokinesis failure, we used an unbiased forward genetic screen using a genome-wide single guide RNA (sgRNA) CRISPR lentiviral library. Taking advantage of the sensitivity of BaF3 cells to cytokinesis failure–induced cell death, we transduced a Cas9-expressing derivative clone with an sgRNA library and exposed cells to Taxol or Taxol in combination with reversine for 48 hours (Fig. 1, A and B). Surviving cells were isolated by Ficoll gradient purification, and the enriched sgRNAs were profiled by next-generation sequencing (NGS). Using a cutoff of at least three of six sgRNAs per gene with a *P* value of <0.05, we identified a series of established genes known to promote mitochondrial apoptosis in cells after Taxol treatment. In addition to p53, sgRNAs targeting *Bim/Bcl2l11*, *Bbc3/Puma*, *Dffb/Cad*, *Itpr2*, *Apaf-1*, and *Casp9* were enriched. In the Taxol + reversine condition, we similarly found apoptotic regulators (*Trp53*, *Bbc3*, *Bax*, *Bim*, *Apaf-1*, and *Casp-9*), as well as key regulators of the centrosome-PIDDosome signaling axis, notably *Cep83/Ccdc41* and *Ankrd26,* in addition to the PIDDosome components *Pidd1/Lrdd, Raidd/Cradd*, and *Casp2* (Fig. 1, C and D, and fig. S2, A and B). A complete list of sgRNAs enriched in our experiment is found in tables S1 and S2.

Consistent with these findings, BaF3 pro-B cells lacking individual PIDDosome components or the key effectors of intrinsic mitochondrial apoptosis, BAX/BAK, were all protected from apoptosis triggered by Taxol + reversine or, alternatively, the Aurora kinase inhibitor ZM. The same genetic knockouts (KOs) also protected BaF3 cells from cell death induced by Taxol only, likely reflecting a poor propensity of these cells to maintain a strong SAC-dependent mitotic arrest over time (Fig. 1E and fig. S2C).

### Caspase-2 triggers mitochondrial outer membrane permeabilization after cytokinesis failure

**Fig. 2.**
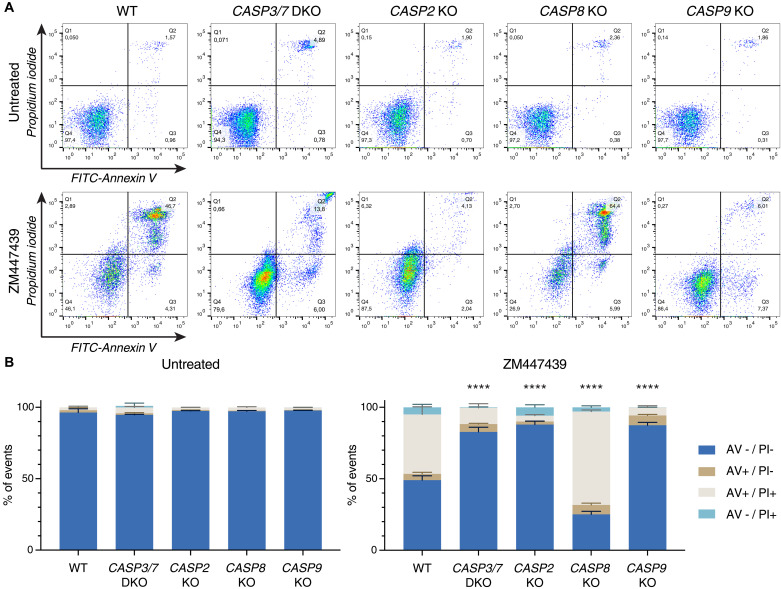
Caspase-2, caspase-9, or combined loss of effector caspase-3/caspase-7 protects Nalm6 cells from cytokinesis failure–induced cell death, but caspase-8 does not. (**A**) Representative dot plots of annexin V/PI–stained Nalm6 WT cells or derivative KO clones after 48 hours of treatment with 2 μM ZM (or untreated controls). (**B**) Quantification of (A). Bar charts represent the means ± SD of the percentage of events in each staining condition. *N* ≥ 3 independent biological replicates. Statistical significance was calculated by unpaired *t* test on the percentage of live cells of each KO derivative clone compared to WT cells. ****P < 0.0001.

Our findings suggest that caspase-2 is activated in the PIDDosome to promote activation of BAX/BAK in hematopoietic cells that fail cytokinesis. For epistasis analysis and to position caspase-2 in the chain of events, we exploited a set of human Nalm6 pre-B ALL cells lacking initiator caspase-8 or caspase-9, as well as cells lacking both effector caspases, caspase-3 and caspase-7, and compared their response to Nalm6 cells lacking caspase-2. Treatment with the Aurora kinase inhibitor ZM, which has been proven selective on Aurora B over Aurora A within living cells (*33*), induced apoptosis following cytokinesis failure most effectively (fig. S1) and was used for further analysis. Cell death was assessed by annexin V/propidium iodide (PI) staining and subsequent flow cytometric analysis. Notably, loss of caspase-2 provided clear protection against cell death, similar to that provided by the loss of caspase-9, or combined loss of caspase-3 and caspase-7. In contrast, deficiency in the initiator caspase-8 failed to do so, excluding a role for death receptor–induced apoptosis in our system (Fig. 2, A and B).

**Fig. 3.**
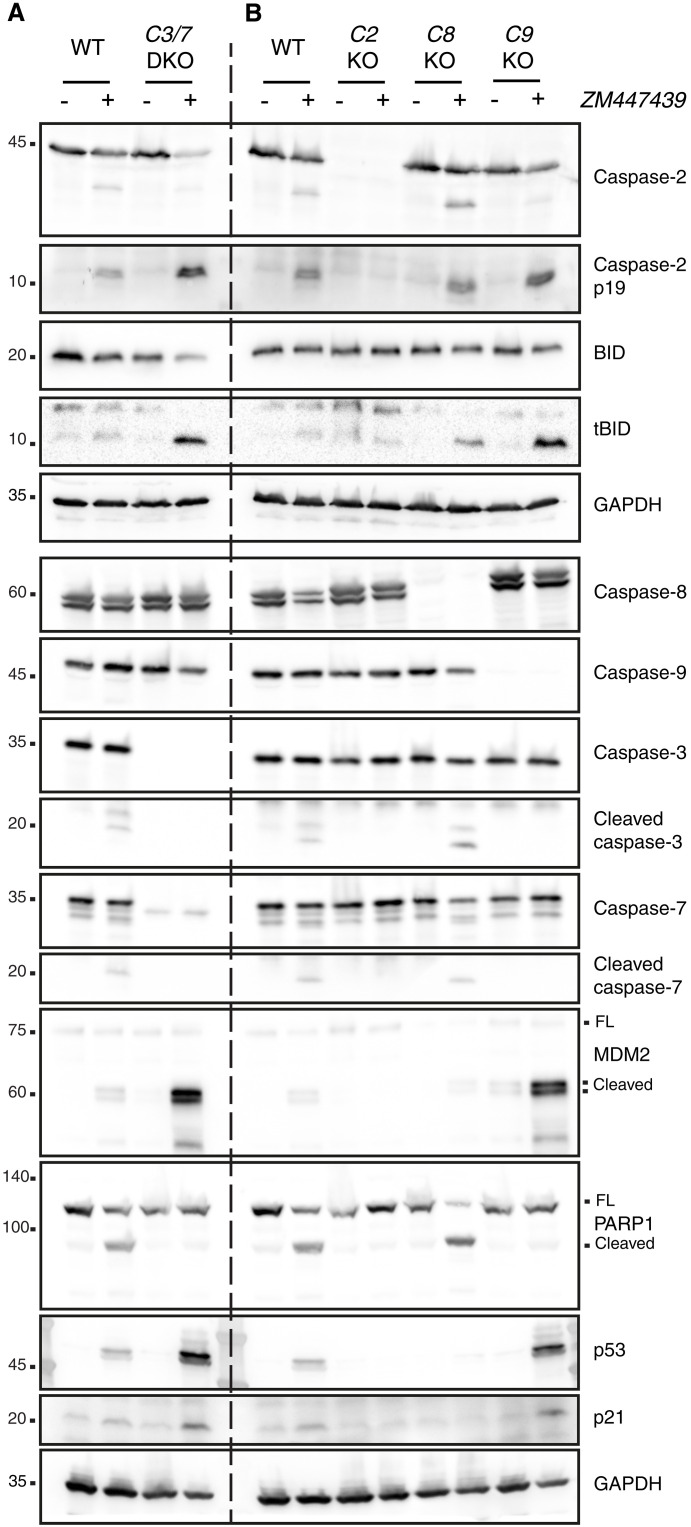
Caspase-2 acts upstream of caspase-9 to trigger apoptosis in Nalm6 cells experiencing cytokinesis failure. (**A**) Western blot analysis of Nalm6 WT and a derivative clone deficient for effector caspase-3/caspase-7 (C3/7 DKO) after 48 hours of treatment with the Aurora kinase inhibitor ZM (2 μM). (**B**) Western blot analysis of Nalm6 WT or KO clones deficient for caspase-2 (C2 KO), caspase-8 (C8 KO), or caspase-9 (C9 KO) after 48 hours of treatment with 2 μM ZM. See also fig. S3.

On the basis of these observations, we wondered whether caspase-2 acts upstream or downstream of caspase-9. To test this, we performed Western blotting analysis to determine caspase activation, and in particular for caspase-2 activation, we used MDM2 processing (which is known to be a substrate of caspase-2) as a readout (*34*). Given its predicted connection to caspase-2–induced cell death, we also monitored for cleavage of the BH3-only protein BID, despite not being a hit in our CRISPR screen. Western blot analysis of parental Nalm6 cells treated with ZM revealed that effector caspase-3 and caspase-7 were processed, indicative of their activation. Moreover, we found accumulation of the processed forms of MDM2, BID, and PARP1, correlating with caspase activation and cell death initiation (Fig. 3A). However, only PARP1 cleavage was blocked in cells lacking effector caspase-3 and caspase-7, while levels of cleaved MDM2, tBID, p53, and p21 were increased (Fig. 3A and fig. S3). This raised the possibility that MDM2 and BID processing occurs upstream of effector caspase activation and hence by an initiator caspase (*35*, *36*).

To confirm if the activation of caspase-2 is the cell death–initiating trigger, we next compared the impact of initiator caspase depletion on MDM2 and BID processing. This comparison revealed that only loss of caspase-2, but not caspase-8 or caspase-9, prevented MDM2 and tBID cleavage, despite similar cell death protection in the absence of caspase-2 or caspase-9 (Fig. 3B). Again, impaired apoptosis in the absence of caspase-9 allowed for increased accumulation of MDM2 cleavage products, p53, p21, and tBID, all of which depended on caspase-2 being present. As indicated before, caspase-8 did not contribute to any of the abovementioned phenotypes (Fig. 3B).

### Selective chemical inhibition of caspase-2 prevents substrate processing and cell death

**Fig. 4.**
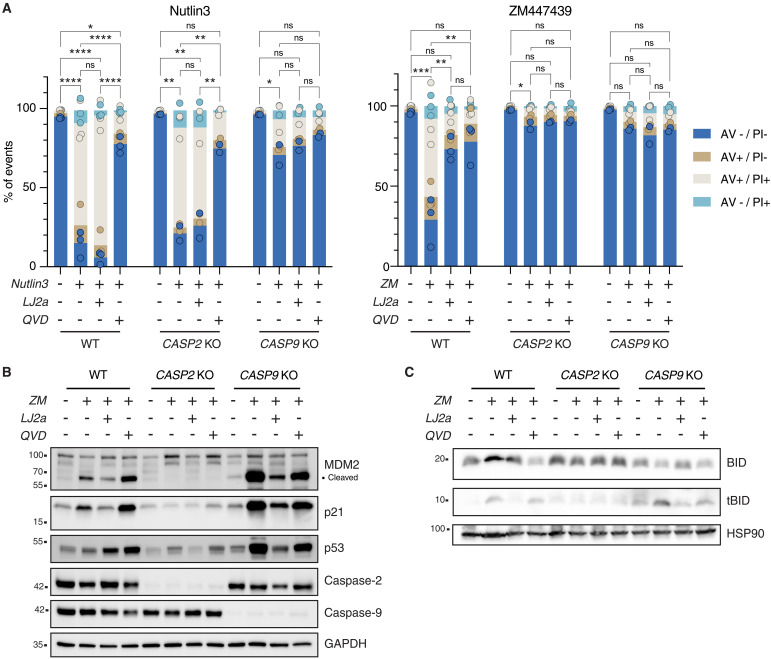
Chemical caspase-2 inhibition reduces cytokinesis failure–dependent cell death. (**A**) Nalm6 WT, caspase-2 KO, and caspase-9 KO cells were treated with 10 μM Nutlin3 or 2 μM ZM alone or in combination with the caspase-2 inhibitor LJ2a (10 μM) or the pan-caspase inhibitor Q-VD-OPh (QVD; 10 μM) or left untreated for 48 hours before staining with annexin V/PI followed by flow cytometric analysis. Bar charts represent the means of the percentage of events in each staining condition, the dots represent the values for each single replicate. Statistical significance was calculated on the percentage of live cells by one-way analysis of variance (ANOVA) with Tukey’s multiple-comparison correction, comparing each condition within the genotype. *N* ≥ 2 independent biological replicates. ns, not significant; *P < 0.05; **P < 0.01; ***P < 0.001; ****P < 0.0001. See also fig. S4. (**B**) Western blot analysis of Nalm6 WT, caspase-2 KO, and caspase-9 KO cells after 48 hours of treatment with 2 μM ZM, alone or in combination with the caspase-2 inhibitor LJ2a (10 μM) or the pan-caspase inhibitor QVD (10 μM) (or untreated controls). (**C**) Western blot analysis of Nalm6 cells as described in (B).

Making use of LJ2a, a recently developed selective caspase-2 inhibitor (*37*), we compared the impact of caspase-2 or pan-caspase inhibition using Q-VD-OPh (QVD) on cell death induced by ZM or the nongenotoxic p53 activator Nutlin3 (*38*). Consistent with the specificity of LJ2a for caspase-2, Nutlin3-induced cell death was abrogated by QVD, but not LJ2a. Cell death in response to Aurora kinase inhibition was prevented by both caspase-2 and pan-caspase inhibition. In support of this view, genetic loss of caspase-9, but not caspase-2, prevented cell death induced by Nutlin3, while both mutants were protected from ZM-induced cell death. The addition of either inhibitor did not provide additional protection to caspase-2 and caspase-9 KO cells failing cytokinesis (Fig. 4A and fig. S4). Western blot analysis corroborated our results using genetic caspase depletion (Fig. 3B) and revealed that cleaved MDM2 accumulated in wild type (WT), but even more so in caspase-9–deleted cells (Fig. 4B). As expected, this was not seen in cells lacking caspase-2. Treatment with QVD protected against cell death in response to cytokinesis failure without preventing accumulation of the MDM2 or BID cleavage products. In contrast to this, LJ2a treatment reduced both MDM2 and BID processing in both WT and caspase-9 KO Nalm6 cells alike (Fig. 4, B and C). Together, this indicates that caspase-2 acts upstream of mitochondria and that this activity can be selectively inhibited by LJ2a and not by QVD. The relative contribution of BID versus MDM2 cleavage to cell death execution remained instead to be established.

### BID is the preferred caspase-2 substrate to promote MOMP 

**Fig. 5.**
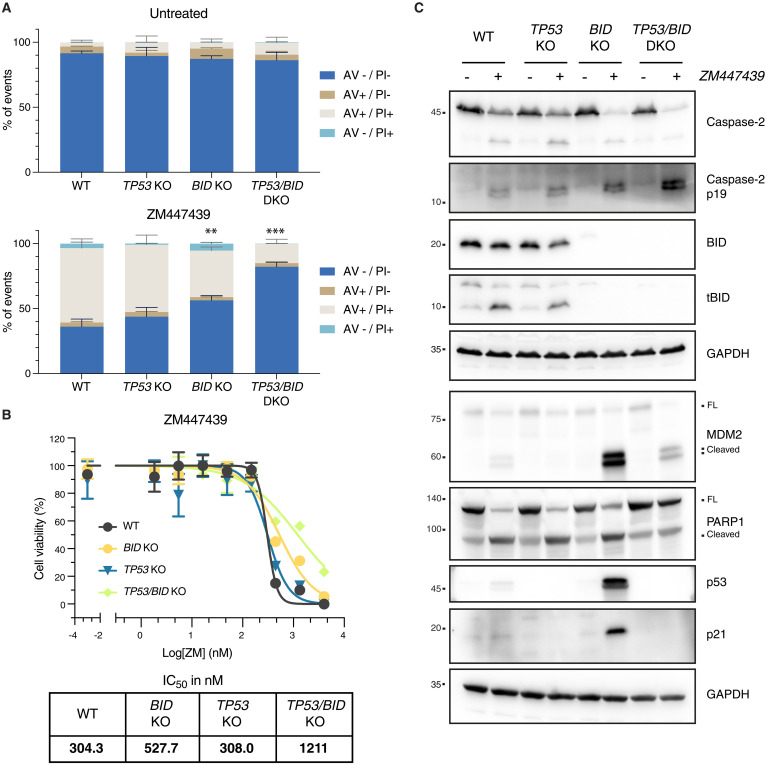
BID and MDM2 act as shared caspase-2 substrates in Nalm6 cells failing cytokinesis. (**A**) Nalm6 WT, BID KO, TP53 KO, or combined TP53/BID (DKO) were treated for 48 hours with 2 μM ZM (or left untreated) before staining with annexin V/PI followed by flow cytometric analysis. Figure S5A reports an example experiment used for the quantifications shown here. Bar charts represent the means and SD of the percentage of events in each condition. Statistical significance was calculated by unpaired *t* test comparing each derivative clone to the WT cells. *N* = 3 independent biological replicates. **P < 0.01; ***P < 0.001. (**B**) Metabolic activity of Nalm6 WT cells and derivative clones deficient for p53, BID, or p53 and BID combined (DKO), as estimated by CellTiter-Glo assay after 72 hours of treatment with the indicated concentrations of ZM. Lines represent the nonlinear regression curves used to calculate the IC_50_ values reported on the bottom right table. (**C**) Western blot analysis of Nalm6 WT and derivative KO clones lacking BID, p53, or the combination of both after 48 hours of treatment with 2 μM ZM.

On the basis of our results, caspase-2 acts upstream of mitochondria by stabilizing p53 via MDM2 cleavage, which may eventually induce transcription of proapoptotic effectors, such as *BBC3/PUMA*. Consistently, both *p53* and *Puma* were identified in our CRISPR screen (Fig. 1). In contrast, *Bid* was not, questioning its actual contribution to mitochondrial outer membrane permeabilization (MOMP) after cytokinesis failure. We therefore anticipated that p53 plays a key role in triggering cell death in response to ZM treatment. To our surprise, loss of p53 in Nalm6 did not protect from ZM-induced killing (Fig. 5A), prompting us to reformulate our hypothesis. Considering that both MDM2 and BID were processed in a caspase-2–dependent manner (Figs. 3B and 4, B and C, and fig. S3C), we assessed whether the two genes are able to compensate for each other. To address this possibility, we generated Nalm6 cells lacking BID or p53 alone, and in combination. Strikingly, while loss of BID alone significantly reduced ZM-induced cell death, combined loss of BID and p53 further enhanced cell death protection (Fig. 5A and fig. S5A). This finding was confirmed in a CellTiter-Glo assay, monitoring metabolic activity as a readout (Fig. 5B). Western blot analysis revealed that BID was processed into tBID following ZM treatment and that MDM2 processing was enhanced in the absence of BID (Fig. 5C). To address whether this enhancement of MDM2 cleavage observed upon BID loss was due to delayed death or rather due to increased capacity of caspase-2 to cleave it, we deleted *BID* and *TP53* in cell death–resistant cells (i.e., devoid of caspase-9). BID loss favored MDM2 cleavage also in these experimental conditions, while loss of p53 (leading to defective MDM2 cleavage) did not alter the extent of tBID production. Together, our data demonstrate that the absence of BID in itself allowed more efficient MDM2 cleavage by caspase-2, independently from longer survival, while lack of MDM2, due to p53 loss, did not alter BID cleavage propensity. Thus, BID is the preferred capsase-2 substrate, while MDM2 cleavage becomes enhanced when BID levels are reduced (fig. S5, B and C).

### Loss of BID or caspase-9 uncovers a PIDDosome-dependent transcriptional p53 response

**Fig. 6.**
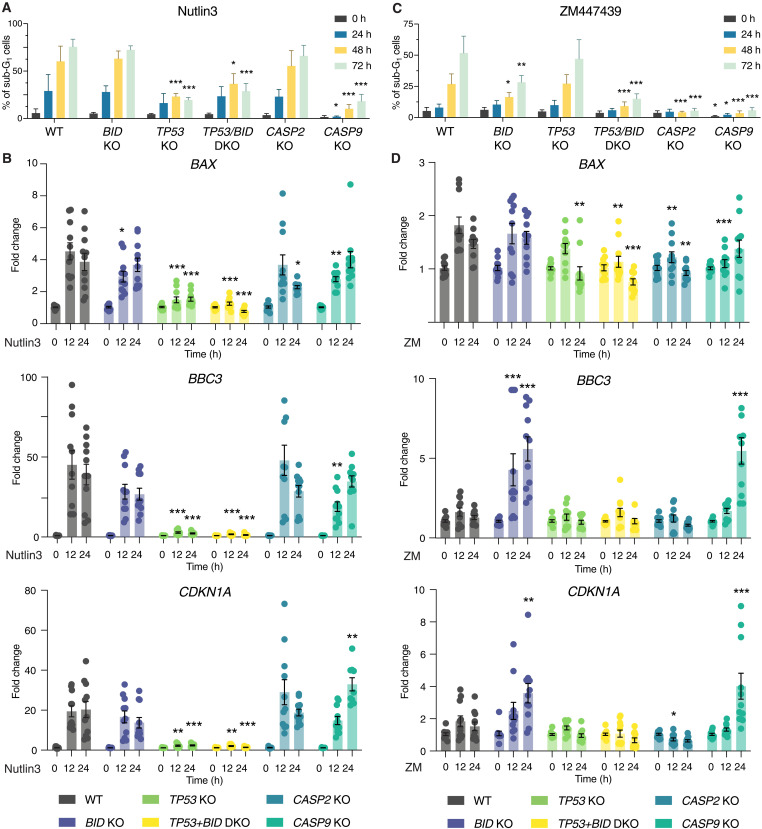
Loss of BID enables p53-dependent transcription in Nalm6 cells after cytokinesis failure. (**A**) Quantification of the percentage of sub-G_1_ cells of different Nalm6 clones at different time points after 10 μM Nutlin3 treatment. Data are presented as means ± SD of *N* ≥ 3 independent biological replicates. Statistical significance was calculated by one-way ANOVA with Dunnett’s multiple-comparison testing, comparing each time point of the KO clones to the corresponding time point of the WT sample. *P < 0.05; **P < 0.01; ***P < 0.001. (**B**) RT-qPCR analysis of the p53 targets BAX, BBC3/PUMA, and CDKN1A/p21 of Nalm6 WT and derivative clones at different time points after 10 μM Nutlin3 treatment. Results are normalized over the housekeeping gene GAPDH and presented as fold change over the time point zero hour for each clone. Data are presented as means ± SEM, and individual points represent the values of *N* = 4 independent biological replicates. Statistical significance was calculated by one-way ANOVA with Dunnett’s multiple-comparison test, comparing each KO clone to the WT sample at the corresponding time point. *P < 0.05; **P < 0.01; ***P < 0.001. (**C**) Same as in (A), but after 2 μM ZM treatment. (**D**) Same as in (B), but after 2 μM ZM treatment.

To better understand the interplay between BID and p53 in cell death initiation in cells failing cytokinesis, we tested different Nalm6 derivative clones lacking distinct cell death regulators after exposure to Aurora kinase inhibitor for cell death induction, ploidy levels, and p53 transcriptional responses. Nutlin3 was again included as a positive control and trigger of a nongenotoxic p53 response. The absence of p53 or caspase-9 effectively protected Nalm6 cells from Nutlin3-induced cell death (Fig. 6A and fig. S6A). As expected, Nutlin3 treatment had no effect on ploidy levels (fig. S6A) but increased the mRNA levels of the established transcriptional p53 targets *BAX, BBC3/PUMA*, and *p21/CDKN1A* across p53-proficient Nalm6 KO cells (Fig. 6B). In contrast, cytokinesis failure via ZM treatment induced cell death in WT and p53 KO cells, but was blunted in BID, caspase-2, and caspase-9 KO cells (Fig. 6C), allowing cells to become highly polyploid (fig. S6B) without a negative impact on cell survival. Remarkably, induction of p53 targets *BBC3/PUMA* and *CDKN1A* was only detectable in cells lacking BID or caspase-9, demonstrating that this PIDDosome-dependent p53 activation is normally masked by the BID-dependent engagement of mitochondrial apoptosis (Fig. 6D).

To test whether this is a more general phenomenon in hematopoietic cells, we created the same set of mutations in BL2 Burkitt lymphoma cells, which corroborated our findings in Nalm6 cells. Again, cell death induced by ZM was dependent on BID and p53 and cells became highly polyploid upon their genetic perturbation (fig. S7, A and B). MDM2 processing as well as p53 accumulation and p21 induction were more prominent in the absence of BID, which itself was effectively processed in WT and p53 mutant cells, as indicated by loss of the full-length fragment (fig. S7D). In line with our findings in Nalm6 cells, transcriptional activation of the p53 targets, *BAX*, *BBC3/PUMA*, and *p21/CDKN1A*, was again strongest on a cell death–refractory BID mutant background (fig. S7C). Nutlin3 treatment induced the expected cell death and transcriptional response without affecting ploidy also in BL2 cells (fig. S7, E to G).

Collectively, our data suggested that, initially, caspase-2 triggers cell death by cleaving BID into tBID to permeabilize mitochondria, yet, when this process is abrogated, a secondary transcriptional p53 response can kick in as a fail-safe mechanism engaging additional apoptotic effectors. Together, our findings assign caspase-2 an apical role in the mediation of p53-induced cell death, reversing the order of events in earlier models (*11*, *39*, *40*).

### Extra centrosomes are required to trigger PIDDosome-dependent apoptosis

**Fig. 7.**
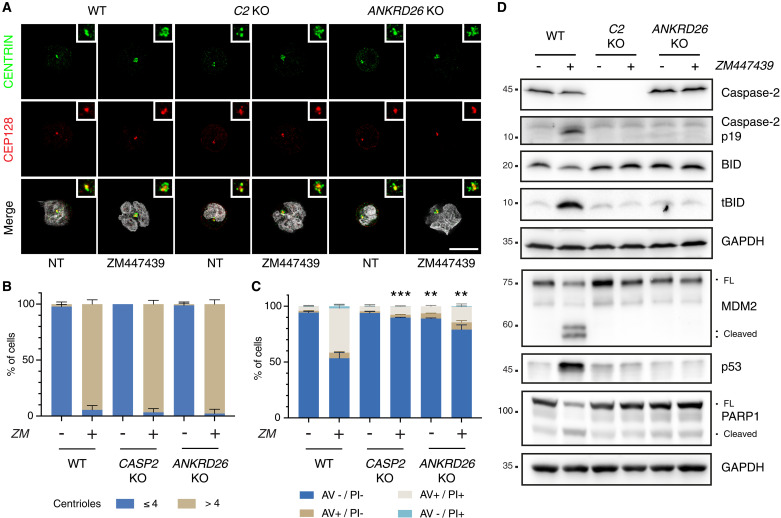
Recruitment of PIDD1 to the centrosome via ANKRD26 is necessary for cytokinesis failure–dependent cell death. (**A**) Representative immunofluorescence of Nalm6 WT, caspase-2, and ANKRD26 KO derivative clones untreated or treated with 2 μM ZM for 48 hours. Cells were costained with the indicated antibodies: CENTRIN (in green) and CEP128 (in red). Hoechst was used to visualize the DNA (gray in the merge). Scale bar, 5 μm. (**B**) Quantification of the number of centrioles of cells shown in (A). Data are presented as means ± SD (in percentage) of three independent biological replicates. For each replicate and condition, 30 cells were counted. (**C**) Percentage of Nalm6 WT, caspase-2 KO, and ANKRD26 KO derivative clones undergoing apoptosis after 48 hours of treatment with 2 μM ZM as detected by annexin V/PI staining and flow cytometric analysis. Data are presented as means ± SD of events in each staining condition (in percentage) of *N* = 3 independent biological replicates. Statistics were calculated by unpaired *t* test comparing the percentage of live cells of each KO clone to the corresponding treatment condition in the WT sample. **P < 0.01; ***P < 0.001. (**D**) Western blot showing Nalm6 WT cells or clones edited for caspase-2 or ANKRD26 after 48 hours of treatment with 2 μM ZM.

We hypothesized that extra centrosomes were the trigger to activate PIDDosome formation and caspase-2 activation in cells that fail cytokinesis. To demonstrate this, we impaired recruitment of PIDD1 to centrosomes by generating Nalm6 cells devoid of the PIDD1 adapter at distal appendages, ANKRD26 (*29*, *30*). Immunofluorescence analysis showed that Aurora kinase inhibition led to a clear accumulation of an aberrantly high number of centrosomes in most cells for all genotypes (Fig. 7, A and B). Consistently with a critical role for extra centrosomes as cell death initiators engaging PIDD1, ANKRD26 mutant cells that failed cytokinesis no longer induced caspase-2 autoprocessing, tBID formation, and MDM2 processing, nor PARP1 cleavage and cell death was strongly reduced (Fig. 7, C and D), phenocopying the findings in caspase-2 mutant cells.

Finally, we assessed the contribution of the ANKRD26-PIDDosome axis to cell death using various drugs perturbing the fidelity of cell division. Strikingly, while a variable contribution of caspase-2 to cell death in mitosis could be appreciated upon all perturbations, the death was ANKRD26 dependent only upon perturbations that bypass the block in mitosis, promoting a direct accumulation of extra centrosomes (fig. S8, A and B). Conceivably, all drugs perturbing mitosis promoted tBID production. Clearly, this appeared to be caspase-2 dependent only upon drug treatments promoting cytokinesis failure (namely, DHCB and ZM). In contrast, tBID production in response to microtubule poisoning with Taxol and nocodazole appeared downstream of MOMP (fig. S8C).

Together, these experiments show that extra centrosomes are the trigger that can activate PIDDosome-dependent mitochondrial apoptosis to limit their own undesired amplification.

### BID overexpression sensitizes epithelial cells to apoptosis upon acquisition of extra centrosomes

**Fig. 8.**
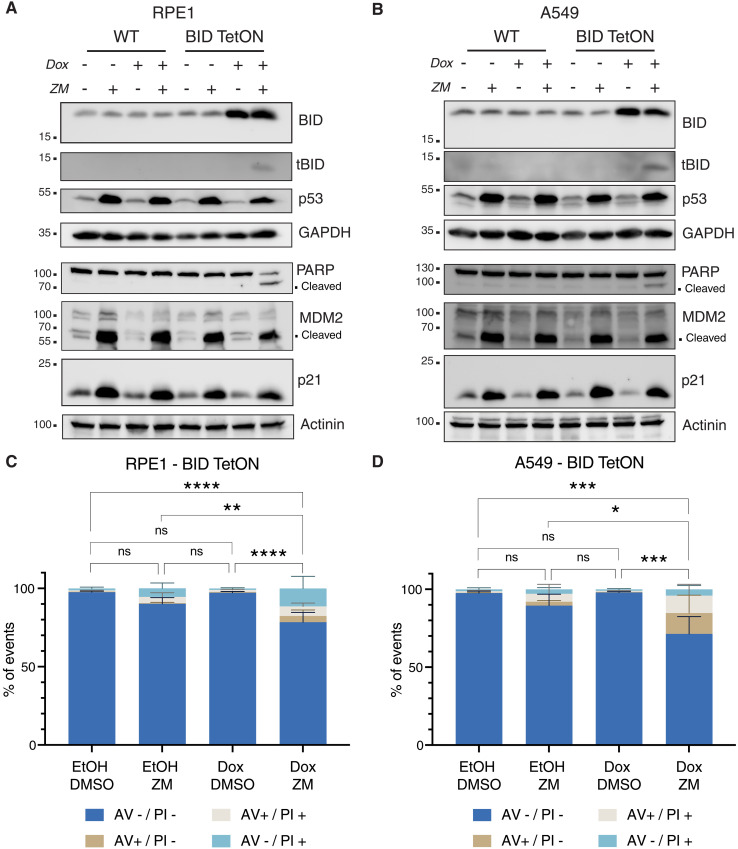
BID overexpression sensitizes epithelial cells to cell death after cytokinesis failure. (**A**) Western blot of RPE1 cells, WT, or a derivative pool transduced with a Dox-inducible BID overexpression vector (BID TetON). Cells were treated for 24 hours with Dox (2.5 μg/ml) or solvent control (ethanol) in combination with 2 μM ZM, or DMSO, as control. (**B**) Same as in (A), but on A549 lung cancer cells. (**C**) Percentage of RPE1 BID TetON undergoing apoptosis upon treatment with 2 μM ZM and overexpression of BID, as detected by annexin V/PI staining in flow cytometric analysis. Bar charts represent the means ± SD of events in each staining condition (in percentage). Statistical significance was calculated by one-way ANOVA with Tukey’s multiple-comparisons test on *N* = 4 independent biological replicates. *P < 0.05; ***P < 0.001; ****P < 0.0001. (**D**) Same as in (C), but in A549 cells.

**Fig. 9.**
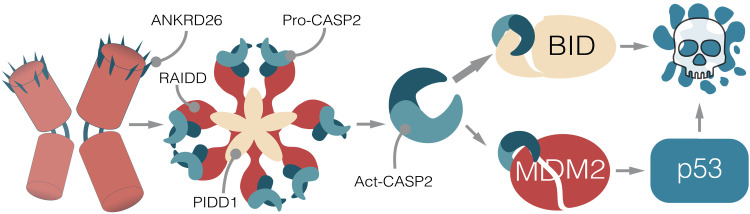
Proposed model of centrosome-dependent cell death. Hematopoietic cells failing cytokinesis acquire extra centrosomes, leading to an ANKRD26-dependent activation of the PIDDosome multiprotein complex. This leads to the autoproteolytic cleavage of caspase-2 into its active form (Act-CASP2), which cleaves BID into its proapoptotic product tBID, leading to MOMP and cell death. If BID processing is prevented, the alternative caspase-2 substrate MDM2 becomes more prominently processed, leading to p53 stabilization. The transcriptional program imposed by p53 will result in the up-regulation of apoptotic effectors, ultimately leading to cell death.

Given that loss of BID per se provided a significant degree of cell death protection in response to supernumerary centrosomes in Nalm6 and BL2 cells, we reasoned that BID expression levels may determine whether epithelial cells, which frequently respond with p53-dependent cell cycle arrest, may eventually commit to apoptosis after cytokinesis failure. This prompted us to test if cell death–resistant RPE1 and A549 epithelial cells can be sensitized to caspase-2–dependent cell death by overexpression of BID. Hence, we generated a doxycycline (Dox)–inducible expression system allowing for BID overexpression and treated cells with the Aurora kinase inhibitor ZM. In line with our hypothesis, upon overexpression, ZM-induced tBID production became evident in both cell lines and the cells became more prone to cell death after cytokinesis failure, as indicated by increased PARP1 cleavage in Western blotting (Fig. 8, A and B) and annexin V/PI positivity (Fig. 8, C and D, and fig. S9). Together, this documents a fate-switch role for BID in the cellular response to cytokinesis failure, as cell death–resistant cell lines, normally responding to cytokinesis failure by displaying p53-p21–dependent cell cycle arrest, can execute some degree of apoptosis as a result of caspase-2–dependent tBID production. A graphical representation of our model is reported in Fig. 9.

## DISCUSSION

For many years, caspase-2 has been implicated in cell death initiation and execution, yet no selective trigger of its action has been identified. Several laboratories, including ours, have aimed to assess the contribution of caspase-2 in the context of apoptosis in response to DNA damage, microtubule poisons, or ER stress (*16*–*18*). Despite having structural features of an initiator caspase, none of these studies has been able to assign an apical position to caspase-2 in any of these cell death modalities. This led to the repeated argument that it acts as a backup or amplifier in canonical, caspase-9–mediated cell death paradigms (*41*, *42*), also downstream of effector caspases (*16*). Moreover, several studies placed caspase-2 downstream of p53 (*39*, *40*, *43*). Its published role in the ill-defined process of “mitotic catastrophe” (*44*, *45*) prompted us to study its role in the context of mitotic perturbations. Building on our previous studies, identifying caspase-2 as an inducer of p53 in the context of centrosome amplification (*8*), we now provide evidence for the ability of extra centrosomes to restrict their own unscheduled duplication by the induction of PIDDosome-driven and caspase-2–dependent apoptosis as the most extreme solution to avoid consequences of potentially pathogenic polyploidy. Several lines of argument support this idea and exclude secondary effects of cytokinesis failure as drivers of cell death. First, different triggers of cytokinesis failure promote caspase-2–dependent mitochondrial apoptosis. Second, our CRISPR screen unbiasedly identified proteins involved in the formation of distal appendages that when lost provided cell death protection, including CEP83 and ANKRD26 itself, that we and the Holland laboratory have recently identified as the PIDD1 adapter at mature centrosomes (*29*, *30*). Finally, loss of ANKRD26 completely abrogated ZM-induced cell death in our hands. Together, this highlights the centrosome as a signaling hub for caspase activation and cell death, able to initiate pyroptosis, downstream of caspase-1 (*46*), and apoptosis as a consequence of caspase-2 activation in the PIDDosome (shown here). Notably, this pro-death role of centrosomes depends on context, e.g., bacterial infection in case of NLRP3 inflammasome activation, as well as their number and cell type, in case of centrosome amplification (discussed below).

The fact that cells acquiring extra centrosomes, for example, in response to cytokinesis failure or defects in the centrosome duplication cycle, tend to lose these structures has been noted but was preferentially explained either by competition of cells that inherit a single centrosome in subsequent pseudo-bipolar cell divisions (*32*) or by the inherent antiproliferative impact on cells carrying extra centrosomes, drastically slowing down proliferation upon p53 activation (*9*). Apoptosis as a consequence of cytokinesis defects or centrosome amplification has also been noted by some, but was never explored mechanistically and often assigned to be a consequence of p53 activation (*47*, *48*). Moreover, the BH3-only protein BID, known for its need to be processed by different endopeptidases (most notably caspase-8) to become active, has almost exclusively been seen as a bridging element between extrinsic death receptor–induced and mitochondrial apoptosis (*27*). As such, a role for BID in other paradigms of intrinsic apoptosis was lacking until now. Our data presented here have several implications. First, caspase-2 can be activated upstream of mitochondria (Fig. 9), settling a longstanding debate in the cell death field (*41*, *49*). Second, caspase-2 processes BID into its active form tBID in a pathophysiological setting unrelated to death receptor signaling to drive MOMP for canonical caspase cascade activation. Also worth mentioning here is that BID has been implicated in the DNA damage response, as its loss reportedly slows down S-phase progression in response to DNA damage (*50*, *51*). This role of BID was seen as highly controversial (*25*) but may be explained by our findings that, in its absence, a more effective p53 response can be mounted in blood cells that accumulate extra centrosomes, a secondary response in cells experiencing DNA damage (*5*, *52*). An interesting view supported by our work relates to the possibility that MDM2 and BID are competing substrates of caspase-2. In support of this idea, Nalm6 and BL2 BID KO cells showed a much stronger accumulation of MDM2 cleavage products in response to ZM treatment when compared to WT cells. In contrast, p53 functional abrogation led to blunted MDM2 cleavage, likely reflecting the inability of MDM2 gene to be transactivated by p53. This perturbation had no impact on the propensity of the cells to cleave BID. Thus, our work supports the notion that BID is the kinetically favored substrate of caspase-2, while MDM2 becomes cleaved as fail-safe mechanism when tBID production is insufficient to trigger MOMP. BID was not a hit of our initial genetic screen in BaF3 cells. This might reflect a false negative of this particular genetic screen or, as alternative, the insufficient BID expression of BaF3 cells.

Our findings also have several implications for targeted therapies currently under preclinical or clinical evaluation. In particular, Aurora kinase inhibitors have shown the most potent effects on cytokinesis across a wide variety of experimental conditions. It is important to note, however, that derangement of the activity of Aurora B kinase activity during cytokinetic abscission can be achieved by any trigger leading to chromosomal missegregation (*53*). Thus, the mechanism described here may be crucial for the effectiveness of a wide variety of compounds targeting mitosis, such as MPS1, CENP-E, or Polo-like kinase inhibitors, in addition to traditional microtubule targeting agents. Hence, assessment of caspase-2 and BID expression levels may help to identify cancers that respond to these therapies. This concept was recently validated in non–small cell lung cancer patients with amplification of Chr22q11, which harbors the *BID* gene locus. Such cells were particularly sensitive to Aurora B or MPS1 kinase inhibition in vitro (*54*). Consistently, BID overexpression rendered A549 and RPE1 cells more susceptible to ZM treatment in our preliminary assays. In the context of cancer treatment, it appears important that loss of p53 provided only minimal protection from ZM-induced killing, raising hopes that the abovementioned drugs may be effective in treating a broad range of tumors independent of their p53 status. This notion is backed by a report implicating induction of the BH3-only protein PUMA in response to Aurora kinase inhibition in p53 proficient and mutated colon cancer lines (*55*). Curiously, p53-independent *PUMA* induction has been suggested to be nuclear factor κB (NF-κB) dependent, but the cue driving it remained undefined. This opens the intriguing possibility that the reported ability of PIDD1 to drive NF-κB activation together with RIPK1 and NEMO in response to centrosome amplification may account for that phenomenon and contribute to cell death initiation in sensitive cell types (*56*). Moreover, the combined loss of BID and p53 provided a cell death protection comparable to loss of caspase-9 or effector caspases. This implies that p53 mutant tumors may rely heavily on BID to undergo apoptosis in the presence of extra centrosomes. Consistently, some mutations in BID that reduce its killing potency have been noted in cancer (*57*), identifying a potential drug resistance mechanism.

Together, the PIDDosome emerges as a multi-pronged signaling platform that is able to instruct cells to either arrest their cell cycle via p53, release secondary messengers to alert the immune system to cells in danger of aneuploidy by engaging RIPK1, or, as the most extreme measure, instruct self-destruction via BID, in settings where cell cycle arrest and senescence may not be suitable to limit cell growth and the resulting malignancy.

## MATERIALS AND METHODS

### CRISPR screen

The murine precursor B cell line BaF3 expressing Cas9 was transduced with the publicly available GeCKO-V2 sgRNA library [Addgene #1000000053 (*58*)] with a multiplicity of infection that resulted in a transduction efficacy of 10 to 30, thereby avoiding expression of multiple sgRNAs per cell. After 24 hours, transduced cells were selected with puromycin (2 μg/ml) and kept in culture for 8 days to allow the sgRNA:Cas9-mediated gene disruption to take place. Cells were treated with Taxol (50 nM) and the combination Taxol (50 nM) and reversine (500 nM) for 48 hours, while a fraction of the untreated population was snap frozen as an untreated control. Surviving cells were isolated via Ficoll density gradient centrifugation (Lympholyte-M Cell Separation Media CL5030) and snap frozen. Generation of virus, transduction of cells, isolation of genomic DNA, polymerase chain reaction (PCR) amplification of sgRNA sequences, and bioinformatic sgRNA enrichment analysis were performed as previously described (*59*). Raw count tables for the sgRNAs are available on Zenodo (DOI: 10.5281/zenodo.13254853).

### Cell culture and drug treatments

A549, hTERT-RPE1, and human embryonic kidney (HEK) 293T cells were cultured in Dulbecco’s modified Eagle’s medium (DMEM) (D5796, Sigma-Aldrich) supplemented with 10% fetal bovine serum (FBS) (F7524, Sigma-Aldrich), penicillin (100 U), and streptomycin (0.1 mg/ml) (P4333, Sigma-Aldrich). BL2 and Ba/F3 cells were cultured in RPMI 1640 (R8758, Sigma-Aldrich) supplemented with 10% FBS, penicillin (100 U), and streptomycin (0.1 mg/ml). Ba/F3 cells were also supplemented with interleukin-3 (IL-3) derived from the filtered supernatant of WEHI-3B cells (*60*).

Nalm6 cells were maintained in RPMI (R8758, Sigma-Aldrich) or Gibco (#31870-025) supplemented with 2 mM l-glutamine (Corning). In either case, media were supplemented with 10% FBS (F7524, Sigma-Aldrich; #1027-106, Gibco) and penicillin-streptomycin solution (P4333, Sigma-Aldrich; #15140-122, Gibco). All cells were grown at 37°C with 5% CO_2_. Cells were routinely tested for mycoplasma contamination by PCR.

Cells were treated with 10 μM Nutlin-3 (10004372, Cayman Chemical), 2 μM ZM (13601, Cayman Chemical; S1103, Selleck Chemicals), 4 μM DHCB (20845, Cayman Chemical), 100 nM nocodazole (13857, Cayman Chemical), 50 nM Taxol (10461, Cayman Chemical), 500 nM reversine (S7588, Selleck Chemicals), 10 μM QVD (HY-12305, MedChem Express; CAY15260-1, Cayman Chemicals), and 10 μM LJ2a (*37*). LJ2a was purchased from Bachem Americas Inc. (Vista, CA, USA) (custom synthesis). All drugs were resuspended in dimethyl sulfoxide (DMSO). Dox (14422, Cayman Chemical) was resuspended in ethanol and used at a final concentration of 2.5 μg/ml.

### Lentiviral-based CRISPR-Cas9 gene KO

Gene KOs in BaF3, Nalm6, and BL2 cells were performed by lentiviral delivery of Cas9 and the sgRNA using the lentiCRISPR v2 backbone (#52961, Addgene; a gift from F. Zhang) (*58*). sgRNAs were cloned into the destination vector following the protocol reported on the Addgene website. The correct insertion of the sgRNA into the vector was confirmed by Sanger sequencing (Microsynth Austria).

Lentiviral vectors were produced by cotransfecting the lentiCRISPR v2 containing the sgRNA with pSPAX2 and VSVg vectors in HEK293T cells using polyethylenimine (PEI; 23966-100, Polysciences Europe GmbH) in a 1 μg:2 μl DNA:PEI ratio. The following day, media were exchanged with fresh DMEM complete, and cells were left for 48 hours. Vectors were harvested by collecting the supernatant, removing debris by centrifugation (500*g*, 5 min), and filtration using 0.45-μm polyethersulfone syringe filters.

Transduction was performed 4 to 24 hours after seeding the cell line of interest by adding one-fourth of the media volume with the supernatant containing the viral vector. For BL2 cell transduction, protamine sulfate (1101230005, Sigma-Aldrich) was added in combination to the viral vectors at a final concentration of 8 μg/ml. Cells were left for 48 hours before starting the selection with puromycin (13884, Cayman Chemical) at a final concentration of 1 μg/ml for BaF3 cells, 1.66 mg/ml for Nalm6 cells, and 1.25 mg/ml for BL2 cells. Media were exchanged every 48 hours, keeping the puromycin selection until the cells in the untransduced well were all dead. Polyclonal pools were expanded, and the gene KO efficiency was tested by Western blotting. To select cells with the highest editing efficiency on BL2 cells, polyclonal pools were seeded on a 96-well format to a concentration of ~5 cells per well and allowed to grow. After expansion, the subpools were tested for KO efficiency via Western blot and only the subpools showing the highest reduction on the protein levels were kept for further experiments. For the pools transduced with constructs targeting TP53, a further selection using 10 μM Nutlin3 for 48 hours was performed to select only for those cells harboring a p53 deletion or mutation. The complete list of sgRNA sequences can be found in table S3.

### RNP-based CRISPR-Cas9 gene KO

Nalm6 KO cell lines were generated using a ribonucleoprotein (RNP)–based CRISPR/Cas9 approach, as outlined by Ghetti *et al.* (*61*) (without the incorporation of a single-strand DNA homology template and NU-7741 treatment). Isogenic clones for all cell lines were obtained through limiting dilution. The presence of gene-disrupting insertion–deletion mutations (INDELs) in edited cells was validated by Sanger sequencing of PCR products encompassing the crispr RNA recognition site, followed by analysis using the Inference of CRISPR Edits (ICE) tool (https://ice.synthego.com) (*62*). crRNA sequences and PCR primers are reported in table S3. Caspase-8, caspase-9, caspase-3,7 [double KO (DKO)], and caspase-3,6,7 [triple KO (TKO)] cell lines were described before (*63*). Caspase-9 p53 KO cells were electroporated as described before. After 2 days, p53 KO cells were enriched by Nutlin-3a treatment (10 μM for 1 week). p53 KO efficiency was verified using the ICE tool. Caspase-9 BID KO cells were generated using BID crRNA 2 as listed in table S3. In this case, single-cell clones were obtained through limiting dilution.

### Cell cycle profiling—Sub-G_1_ analysis

Cells were collected at the desired time point after the treatment and washed once with phosphate-buffered saline (PBS) before fixation in 70% cold ethanol and stored for at least 4 hours at −20°C.Subsequently, fixed cells were pelleted (1000*g*, 3 min) and washed twice in PBS before resuspension in DNA staining solution, composed of PI (10 μg/ml) (14289, Cayman Chemical) and ribonuclease (RNase) A (100 μg/ml) (10109169001, Roche) in PBS. The volume of DNA staining solution varied depending on the size of the cell pellet, to not exceed a concentration of 1 × 10^6^ cells/ml. Samples were filtered using 50-μm Filcon (340631, BD Biosciences) and acquired using an LSR Fortessa Cell Analyzer (BD Biosciences). Analysis was performed using FlowJo v10, setting the gates to remove doublets before determining the threshold for the sub-G_1_ population.

### Cell viability assay

Nalm6 cells were seeded in 96-well plates at a density of 5000 cells per well, incubated for 24 hours, and subsequently treated with DMSO or ZM. The experiments were performed in biological triplicates, and ZM was serially diluted to 4000, 1333, 444.4, 148.1, 49.39, and 16.46 nM before cell treatment. Cells were then incubated for 72 hours, and the adenosine triphosphate (ATP) content of every well was assessed on the Plate Reader Platform Victor X3 model 2030 (PerkinElmer) using the CellTiter-Glo assay (Promega G7573), following the manufacturer’s protocol. Data were analyzed using GraphPad Prism v10. All data points were normalized to every cell line’s respective mean luminescence DMSO control. Dose-response curves were generated by nonlinear regression curve fitting from which then IC_50_ (median inhibitory concentration) values were derived.

### RNA extraction, cDNA conversion, and RT-qPCR

Cells were collected after treatment and washed once with PBS before snap freezing. Pellets were resuspended in 100 μl of PBS before adding an equal volume of TRIzol reagent (15596026, Invitrogen). After 5 min of incubation, chloroform was added (one-fifth of TRIzol volume) and samples were vortexed for a few seconds and incubated for 3 min before centrifugation (12,000*g*, 4°C, 15 min). The clear upper phase was collected and transferred to a new tube and added with 0.5 ml of isopropanol and 1 μl of GlycoBlue Coprecipitant (AM9516, Invitrogen) before the next centrifugation step (12,000*g*, 4°C, 10 min). The supernatant was removed, and the RNA pellet was washed once with 75% ethanol. After another centrifugation step (12,000*g*, 4°C, 10 min), ethanol was removed and the pellet was left to dry for 5 min before resuspension in molecular biology–grade water (46-000-CV, Corning). RNA concentration was determined using NanoDrop 2000c (Thermo Fisher Scientific). Depending on the sample concentration, 750 to 1500 ng of RNA were used for retrotranscription to cDNA. cDNA conversion was performed using the RevertAid First Strand cDNA Synthesis Kit (K1622, Thermo Fisher Scientific) according to the manufacturer’s instructions. Samples were diluted with molecular biology–grade water to a final concentration of 10 ng/μl, assuming 100% retrotranscription efficiency.

Reverse transcription quantitative PCR (RT-qPCR) was performed starting from 20 ng of cDNA for each target gene and in technical duplicate or triplicate using the qPCRBIO SyGreen Blue Mix (PB20.17-20, PCR Biosystems) following the manufacturer’s instruction. The complete list of primers used for RT-qPCR can be found in table S3. Amplification and detection were performed using CFX Opus 384 Dx (Bio-Rad), and *C*_t_ values were determined using CFX Maestro 2.3 (Bio-Rad). Fold changes were calculated over the housekeeping gene and the corresponding control treatment using the 2^−ΔΔCt^ method using Microsoft Excel. Plots were produced using Prism v10 (GraphPad).

### Western blot

After treatment with the desired drug (or DMSO control), cells were collected and washed once with PBS and pellets were snap frozen. An appropriate volume of Lysis Buffer [50 mM tris-HCl (pH 7.4), 150 mM NaCl, 0.5 mM NP-40, 50 mM NaF, 1 mM Na_3_VO_4_, 1 mM phenylmethylsulfonyl fluoride, deoxyribonuclease I (30 μg/ml) (DN25-10MG, Sigma Aldrich), 1 tablet/10 ml of cOmplete protease inhibitor, EDTA-free (4693132001, Roche)] was added to the cell pellet for 40 min before centrifugation (16,000*g*, 4°C, 12 min). Supernatants were collected and quantified using Pierce BCA Protein assay (23227, Thermo Fisher Scientific). Depending on the sample quantification and the proteins to be detected, a volume corresponding to 20 to 60 μg of proteins was used for SDS–polyacrylamide gel electrophoresis using tris/glycine/SDS buffer. Blotting was performed in wet conditions using a tris/glycine buffer added with 20% ethanol on nitrocellulose membranes (GE10600002, Cytiva). Polyvinylidene difluoride (GE10600029, Cytiva) membranes were used for the detection of tBID. Blocking was performed in 5% milk (T145.3, Carl Roth) in Tris-buffered saline with 0.1% Tween® 20 detergent (TBS-T). Antibodies were all diluted in 1% milk in TBS-T (complete list is reported in table S3); primary antibodies were all incubated overnight at 4°C, whereas secondary antibodies were incubated for 1 hour at room temperature. Detection was performed by incubating the membranes with ECL Select Western Blotting Detection Reagent (GERPN2235, Cytiva) for 3 min before detection using a ChemidocMP (Bio-Rad) or Alliance LD2 Imaging System (UviTec Cambridge).

### Immunofluorescence

Cells, treated or untreated, were counted, and 100,000 cells were plated on 12-mm glass coverslips precoated with poly-d-lysine (100 μg/ml). Then, cells were left for 30 min at room temperature or 4°C and fixed and permeabilized with absolute ice-cold methanol for at least 20 min at −20°C.

Cells were rinsed with PBS, blocked with 3% (w/v) bovine serum albumin in PBS for 20 min, and stained for 1 hour at room temperature with primary antibodies diluted in blocking solution. Cells were washed with PBS and incubated with fluorescent secondary antibodies for 45 min at room temperature. DNA was stained with Hoechst 33342 (1 μg/ml) (Invitrogen). After incubation, cells were rinsed with PBS and double-distilled water, and mounted using ProLong Gold Antifade Reagent (Invitrogen). The list of antibodies is reported in table S3.

Images for quantification were acquired using a Nikon Eclipse Ti2 inverted microscope, equipped with a CrestOptics X-Light V2 spinning disc module, a Lumencor SpectraX light engine, and an Andor iXon Ultra 888 electron multiplying charge-coupled device camera using a Plan Apochromatic 100×/1.45 oil immersion objective. Representative images were acquired on a Leica TCS SP8 microscope using a 63×/1.4 oil objective with Lightening mode (adaptive as “Strategy” and ProLong Gold as “Mounting medium”) to generate deconvolved images. Images were processed using Fiji and displayed as maximum intensity projections of deconvolved z-stacks.

### Annexin V–PI staining

Cells were collected after the treatments and washed twice with PBS before resuspension in 100 μl of Annexin Binding Buffer (140 mM NaCl, 2.5 mM CaCl_2_, 10 mM Hepes, pH 7.4). Samples were added with 5 μl of fluorescein isothiocyanate (FITC) annexin V antibody (640906, BioLegend) and 10 μl of PI (0.5 mg/ml). Alternatively, samples were stained using the FITC Annexin V Apoptosis Detection Kit I (BD Pharmingen, 556547) following the manufacturer’s instructions. After vortexing, samples were incubated for 15 min at room temperature in the dark before adding 400 μl of Annexin Binding Buffer and proceeding with the acquisition using an LSR Fortessa Cell Analyzer (BD Biosciences) or a Symphony A1 cytometer (BD Biosciences). Data were processed using FlowJo v10 (FlowJo LLC), and summary plots were obtained using Prism 10 (GraphPad).

### BID overexpression

BID sequence was amplified from hTERT-RPE1 cell cDNA using primers containing the restriction sites for Nhe I and Age I (primer sequence reported in table S3). The PCR fragment as well as the destination vector pCW57.1 (Addgene plasmid #41393, a gift from D. Root) were digested using Nhe I–HF (R3131S, NEB) and Age I–HF (R3552S, NEB). The PCR fragment was subsequently purified using the Wizard SV Gel and PCR Clean-Up System (A9281, Promega) according to the manufacturer’s instructions. The vector was dephosphorylated using rSAP (M0371S, NEB) and loaded on a gel, and the correct band was purified using Wizard SV Gel and PCR Clean-Up System. Ligation of the PCR product with the purified destination vector was performed using T4 DNA Ligase (M0202S, NEB) and transformed in Stbl3 bacteria. Minipreps from selected colonies were sequenced by Sanger sequencing (Microsynth Austria) to ensure the correct cloning of the insert.

Viral vectors and transduction were performed as described above (see the “Lentiviral-based CRISPR-Cas9 gene KO” section), with the only exception of plasmid pMD2.G (Addgene plasmid #12259, a gift from D. Trono) being used instead of VSVg. A549 cells were selected with puromycin (2.5 mg/ml) and hTERT-RPE1 with puromycin (10 mg/ml) and kept in selection until control cells were all dead. After expansion, the pools of transduced cells were cotreated with Dox (2.5 μg/ml) (Cay14422-1, Cayman Chemical) or the corresponding volume of ethanol and ZM for 24 hours before collection and prepared for Western blot analysis. For annexin V/PI staining, A549 and RPE1 cells were cotreated with Dox (or ethanol) and ZM (or DMSO) 24 hours after seeding.

### Statistical analysis

Data are presented as means and SD or SEM as reported in the legends for each panel. All the statistical analyses were performed using GraphPad Prism v10 (GraphPad Software). Details on the statistical tests, number of replicates, and significance intervals are reported in the figure legends.
