# Supplementary material for: Caspase-2 kills cells with extra centrosomes
Source: Sci Adv. 2024 Oct 30;10(44):eado6607. doi: 10.1126/sciadv.ado6607 (PMC11524169; doi:10.1126/sciadv.ado6607)
Supplement: Supplementary file 1 — Figs. S1 to S9 Legends for tables S1 to S3 [file sciadv.ado6607_sm.pdf]

Science Advances
AAAS

Supplementary Materials for

Caspase-2 kills cells with extra centrosomes

Dario Rizzotto *et al.*

Corresponding author: Andreas Villunger, andreas.villunger@i-med.ac.at; Luca L. Fava, luca.fava@unitn.it

Sci. Adv. **10**, eado6607 (2024)
DOI: 10.1126/sciadv.ado6607

The PDF file includes:

Figs. S1 to S9
Legends for tables S1 to S3

Other Supplementary Material for this manuscript includes the following:

Tables S1 to S3

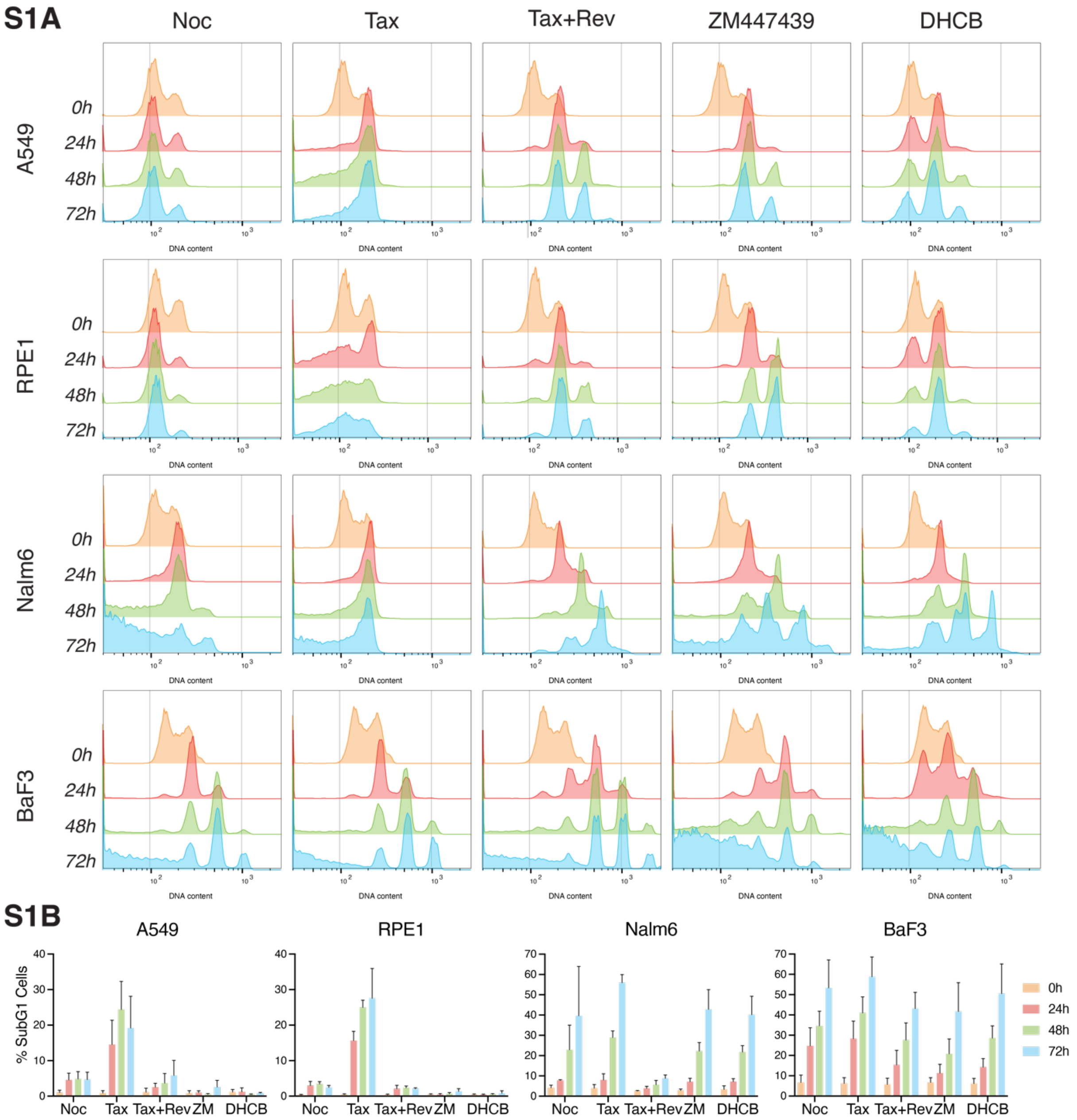

Fig. S1: The outcome of mitotic errors is cell line dependent. (A) Representative DNA content profiles of A549, RPE1, Nalm6 and BaF3 cell lines exposed to different drugs interfering with mitotic progression (100nM Nocodazole, Noc; 50nM Taxol, Tax) and cytokinesis (50nM Taxol + 500nM Reversine, Tax+Rev; 2µM ZM447439, 4µM DHCB) as detected by propidium iodide staining and flow cytometric analysis. For each treatment, three different time points were analyzed. **(B)** Quantification of the subG1 events from A. Data is presented as mean ± SD of N ≥ 3 independent biological replicates for each condition.

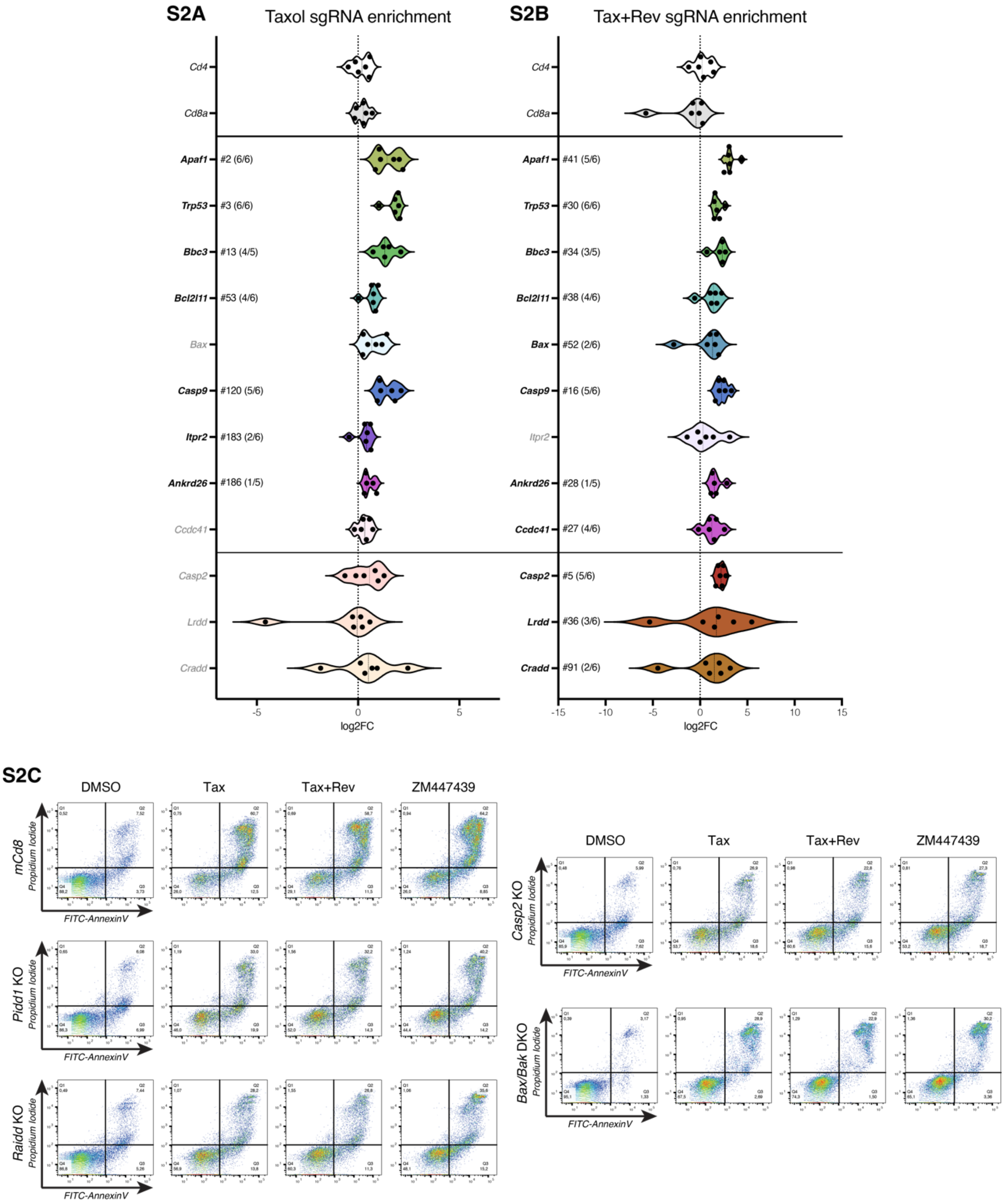

Fig. S2: BaF3 CRISPR Screen sgRNA distribution for selected genes and validation of the central role of the PIDDosome in mitotic error-induced apoptosis. (A) log2 fold change (log2FC) distribution of significantly enriched sgRNA targeting Trp53 and other genes involved in apoptosis after Taxol treatment (50nM). Each dot represents a sgRNA. Cd4 and Cd8a were included as negative controls. Genes marked in bold are significantly enriched (p value < 0.05). The number following the # indicates enrichment ranking of the gene whereas the values in brackets indicate the number of enriched sgRNAs targeting that gene. **(B)** log2 fold change distribution of significantly enriched sgRNA targeting the PIDDosome components, Trp53 and genes involved in apoptosis after Taxol+Reversine treatment (50nm

+ 500nM). Each dot represents a sgRNA. Cd4 and Cd8a were included as negative controls. Genes reported in bold are significantly enriched (p value < 0.05). The number following the # indicates gene enrichment ranking whereas the values in brackets indicate the number of enriched sgRNAs targeting that gene. **(C)** Representative dot plot examples of flow cytometric AnnexinV/PI analyses of BaF3 cells lacking Pidd1, Raidd, Casp2, Bak and Bax, or harboring a control guide RNA targeting mouse Cd8 (mCd8). Cells were treated for 48 hours with 50nM Taxol, 50nM Taxol + 500nM Reversine and 2µM ZM447439. Quantification is shown in Fig.1E.

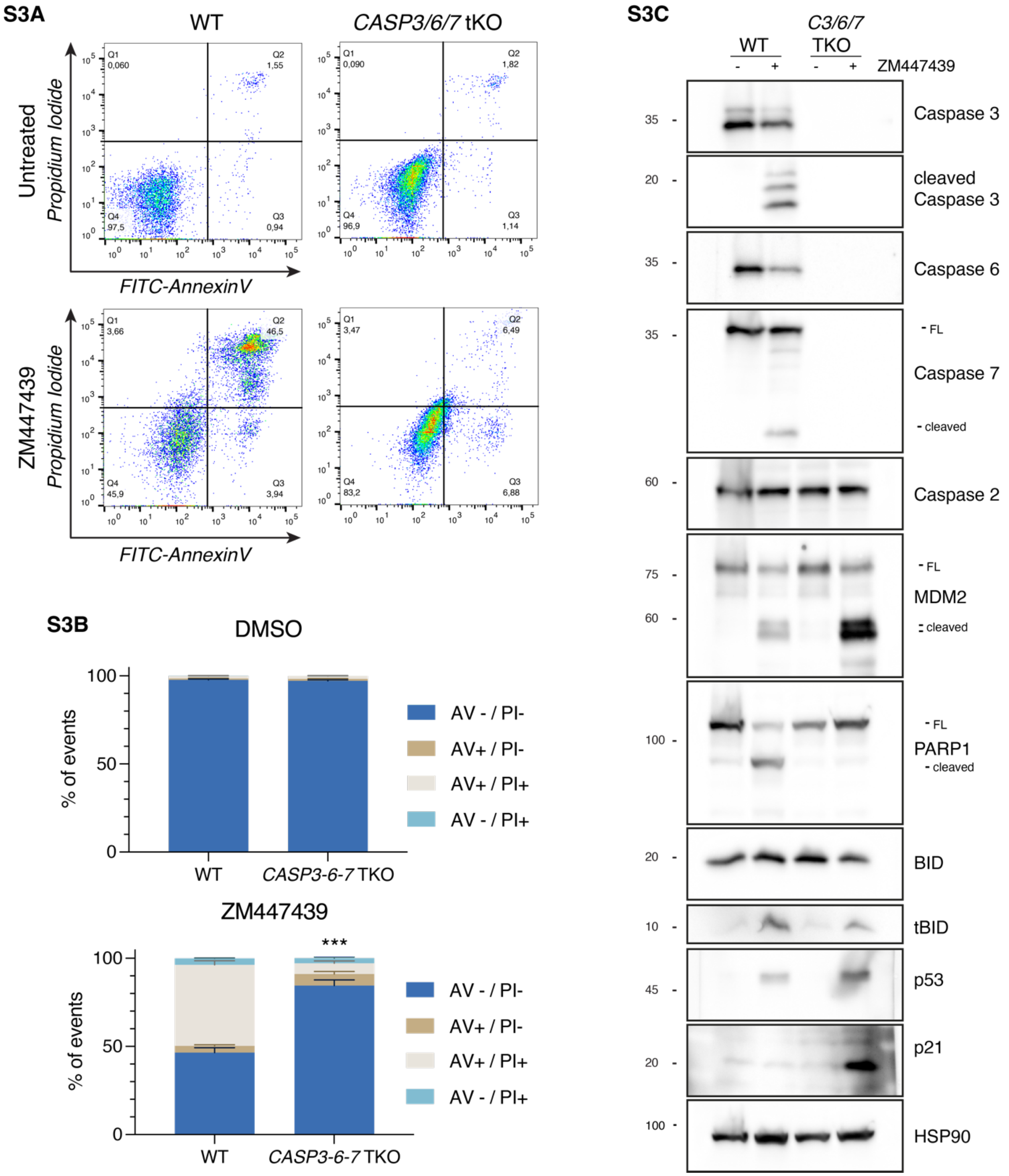

Fig. S3: Caspase-3,-6,-7 triple KO cells show enhanced survival and increased MDM2 processing after ZM447438 treatment. (A) Representative dot-plots of AnnexinV/PI stained Nalm6 WT cells and a derivative clone lacking effector caspases-3,-6,-7 after 48h of treatment with 2uM ZM447439 (or untreated controls). **(B)** Quantification of A. Bar charts represent the means ± SD of the percentage of events in each staining condition. N = 3 independent biological replicates. Statistical significance was calculated by unpaired t test on the percentage of live cells of the caspase-3,-6,-7 triple KO clone compared to WT cells. *** = p value < 0.001 **(C)** Western blot analysis of WT and effector caspases-3,-6,-7 TKO cells after 48h of treatment with the Aurora kinase inhibitor ZM447439 (2µM).

S4A

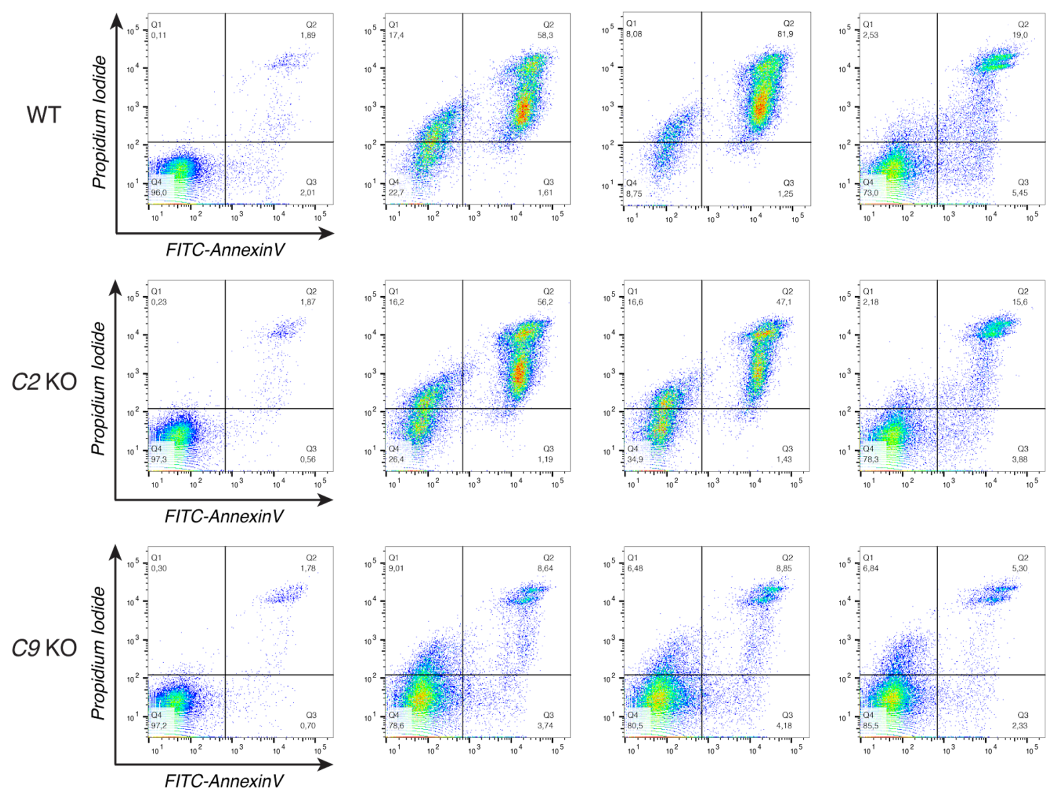
Untreated
Nutlin3
Nutlin3+LJ2a
Nutlin3+QVD
WT
C2 KO
C9 KO
Propidium Iodide
FITC-AnnexinV

S4B

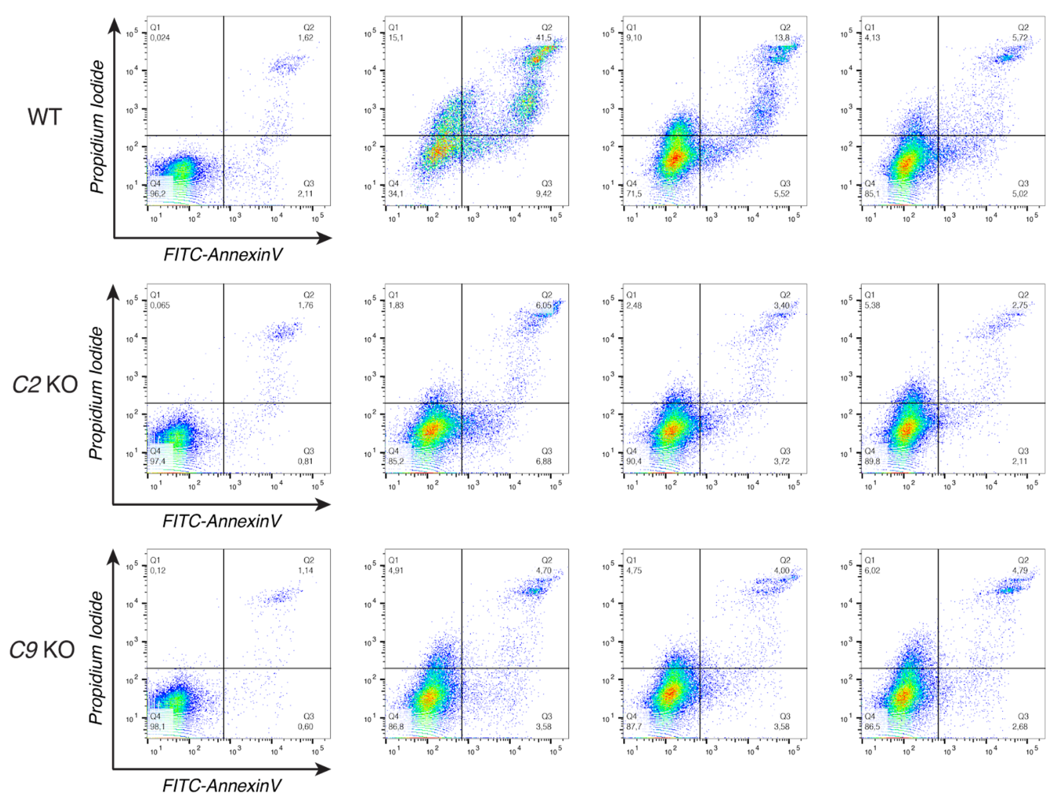
Untreated
ZM
ZM+LJ2a
ZM+QVD
WT
C2 KO
C9 KO
Propidium Iodide
FITC-AnnexinV

Fig. S4: Loss or inhibition of caspase-2 prevents only cytokinesis failure-dependent apoptosis but not Nutlin3-induced cell death. Representative dot plots examples of AnnexinV/PI staining of Nalm6 WT cells or derivative clones edited for caspase-2 or caspase-9 after 48 hours of treatment with 10µM Nutlin3 (panel A) or 2µM ZM447439 (panel B) alone or in combination with the caspase-2 inhibitor LJ2a (10µM) or the pan-caspase inhibitor QVD (10µM). Quantification is shown in Fig. 4A.

S5A

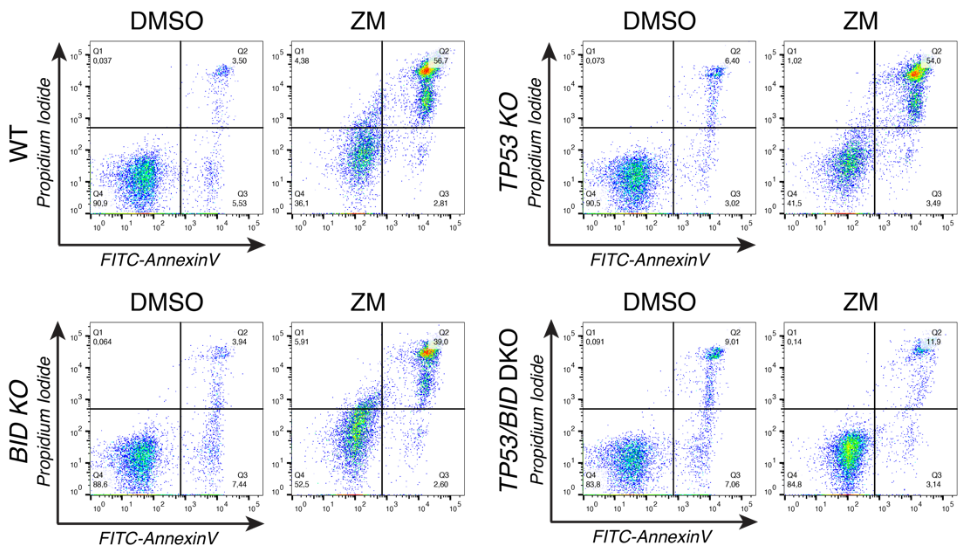

DMSO
ZM
DMSO
ZM
WT
Propidium Iodide
FIT C-AnnexinV
TP53 KO
Propidium Iodide
FITC-AnnexinV
BID KO
Propidium Iodide
FITC-AnnexinV
TP53/BID DKO
Propidium Iodide
FITC-AnnexinV

S5B

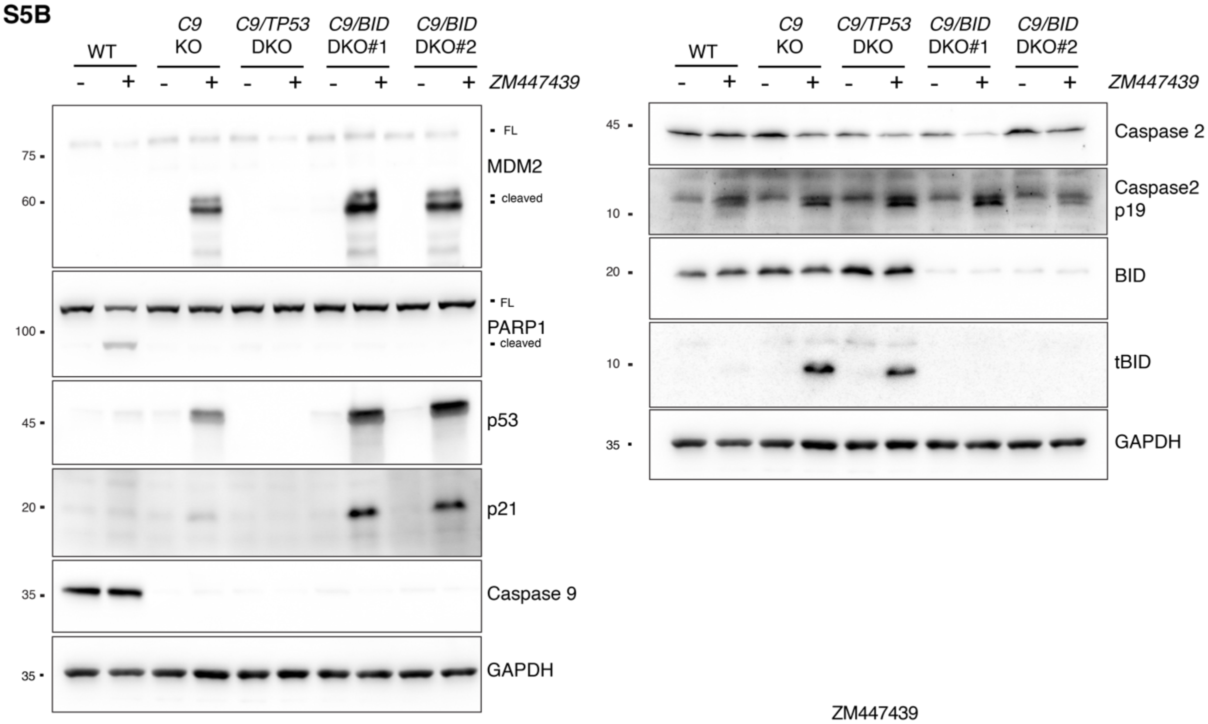

WT
C9 KO
C9/TP53 DKO
C9/BID DKO#1
C9/BID DKO#2
ZM447439
75
60
FL
MDM2
cleaved
100
FL
PARP1
cleaved
45
p53
20
p21
35
Caspase 9
35
GAPDH
45
Caspase 2
10
Caspase2 p19
20
BID
10
tBID
35
GAPDH

S5C

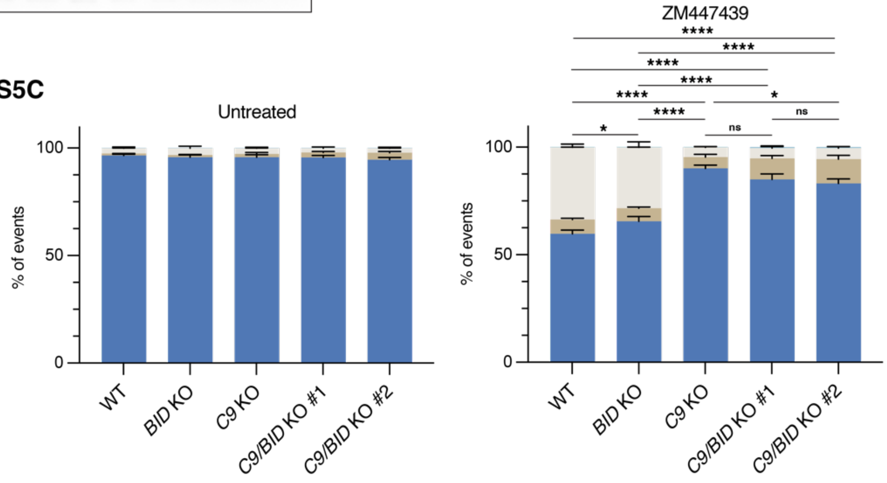

Untreated
ZM447439
% of events
100
50
0
WT
BID KO
C9 KO
C9/BID KO #1
C9/BID KO #2
****
*
ns

Fig. S5: BID is the kinetically favored caspase-2 substrate. (A) Representative dot-plots of AnnexinV/PI staining of Nalm6 WT cells or derivative clones lacking p53, BID or both after 48 hours of treatment with 2µM ZM447439. Quantification is shown in Fig. 5A. **(B)** Western blot of Nalm6 WT and derivative clones lacking caspase-9 only, caspase-9 in combination with p53 (C9/TP53 DKO) or BID (C9/BID DKO, two independent clones #1 and #2) after 48h of treatment with ZM447439 (ZM, 2µM). **(C)** Quantification of AnnexinV/PI staining and flow cytometric analysis of Nalm6 WT and derivative clones described in B treated for 48h with 2µM ZM447439. Data are presented as means ± SD of the percentage of events in each condition. N = 3 independent biological replicates. Statistical significance was calculated by one-way ANOVA with Tukey's multiple comparison test on the percentage of live cells within each genotype. ns = not significant; * = p value < 0.05; **** = p value < 0.0001.

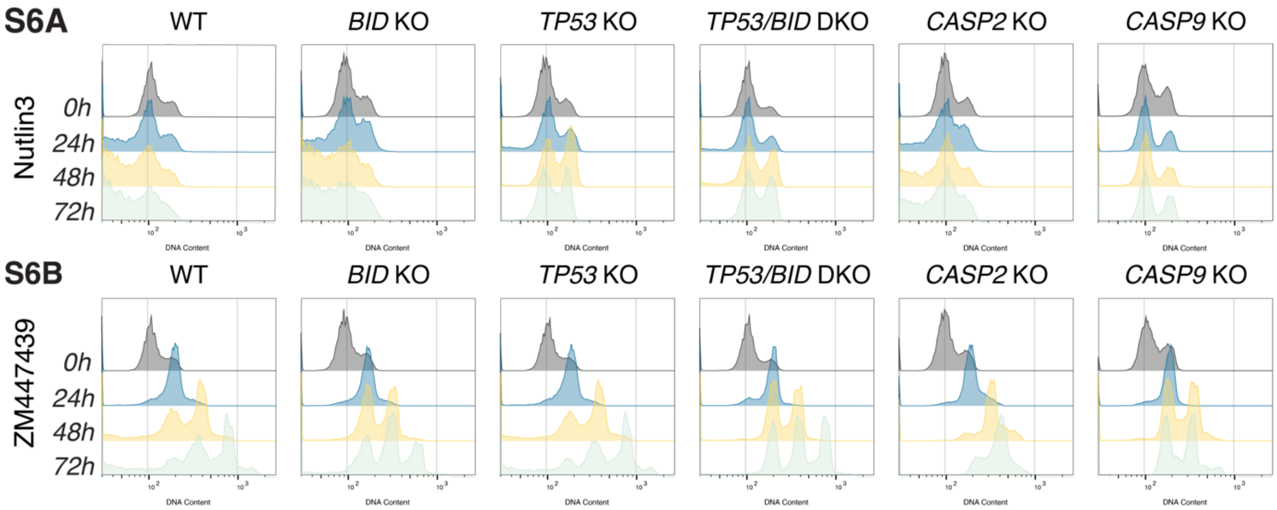

Fig. S6: Combined loss of BID and p53 impairs cytokinesis failure-induced cell death. (A) Representative DNA content profiles of Nalm6 WT cells and derivative clones at different time points after 10µM Nutlin3 treatment. Quantification of the subG1 events shown in Fig. 6A. **(B)** Same as in A, after cytokinesis failure induced by 2µM ZM447439. Quantification of the subG1 events shown in Fig. 6C.

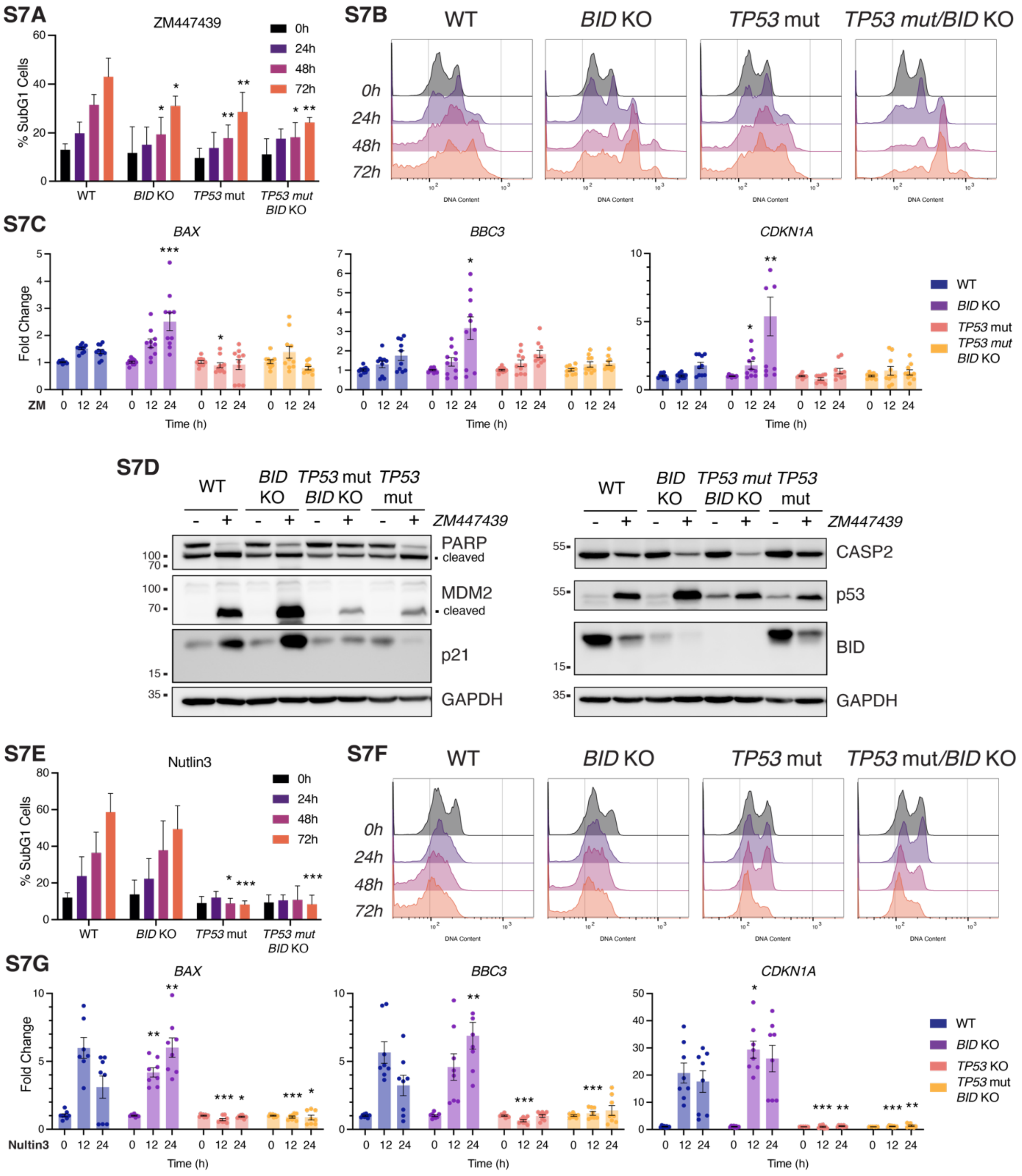

Fig. S7: BID and p53 cooperate to induce apoptosis in the p53 proficient Burkitt lymphoma cell line BL2 after cytokinesis failure. (A) Quantification of the percentage of subG1 BL2 cells WT and derivative CRISPR/Cas9 edited pools harboring sgRNAs targeting BID, p53 or both at different time points after treatment with 2µM ZM447439. Data is presented as means ± SD of N ≥ 4 independent replicates. Statistics was calculated by one-way ANOVA with Dunnett's multiple comparison test, comparing each time point of the KO pool to the corresponding time point in the WT sample. * = p value < 0.05; ** = p value < 0.01; ** = p value < 0.001. **(B)** Example of the DNA content profiles of BL2 WT and derivative pools transduced with lentiCRISPR constructs targeting BID, p53 or both in combination at different time points after treatment with 2µM ZM447439. SubG1 events are quantified in Fig. S6C. **(C)** RT-qPCR

analysis of the p53 targets BAX, BBC3/PUMA and CDKN1A/p21 on BL2 cells at different time points after 2µM ZM447439 treatment. Results are normalized over the house- keeping gene GAPDH and presented as fold-change over the time point 0h for each polyclonal cell line pool. Data is presented as means ± SEM and individual points represent the value of the independent biological replicate (N = 4). Statistical significance was calculated by one-way ANOVA with Dunnett's multiple comparison test, comparing each time point of a KO clone to the corresponding time point of the WT sample. * = p value < 0.05; ** = p value < 0.01; *** = p value < 0.001. **(D)** Western blot analysis of BL2 cells and derivative polyclonal pools transduced with lentiCRISPR constructs targeting BID, p53 or both in combination after 48h of treatment with 2µM ZM447439. **(E)** Same as in A but after 10µM Nutlin3 treatment. N ≥ 2 independent biological replicates. **(F)** Same as in B but after 10µM Nutlin3 treatment. **(G)** Same as in C but after 10µM Nutlin3 treatment. N = 3 independent replicates

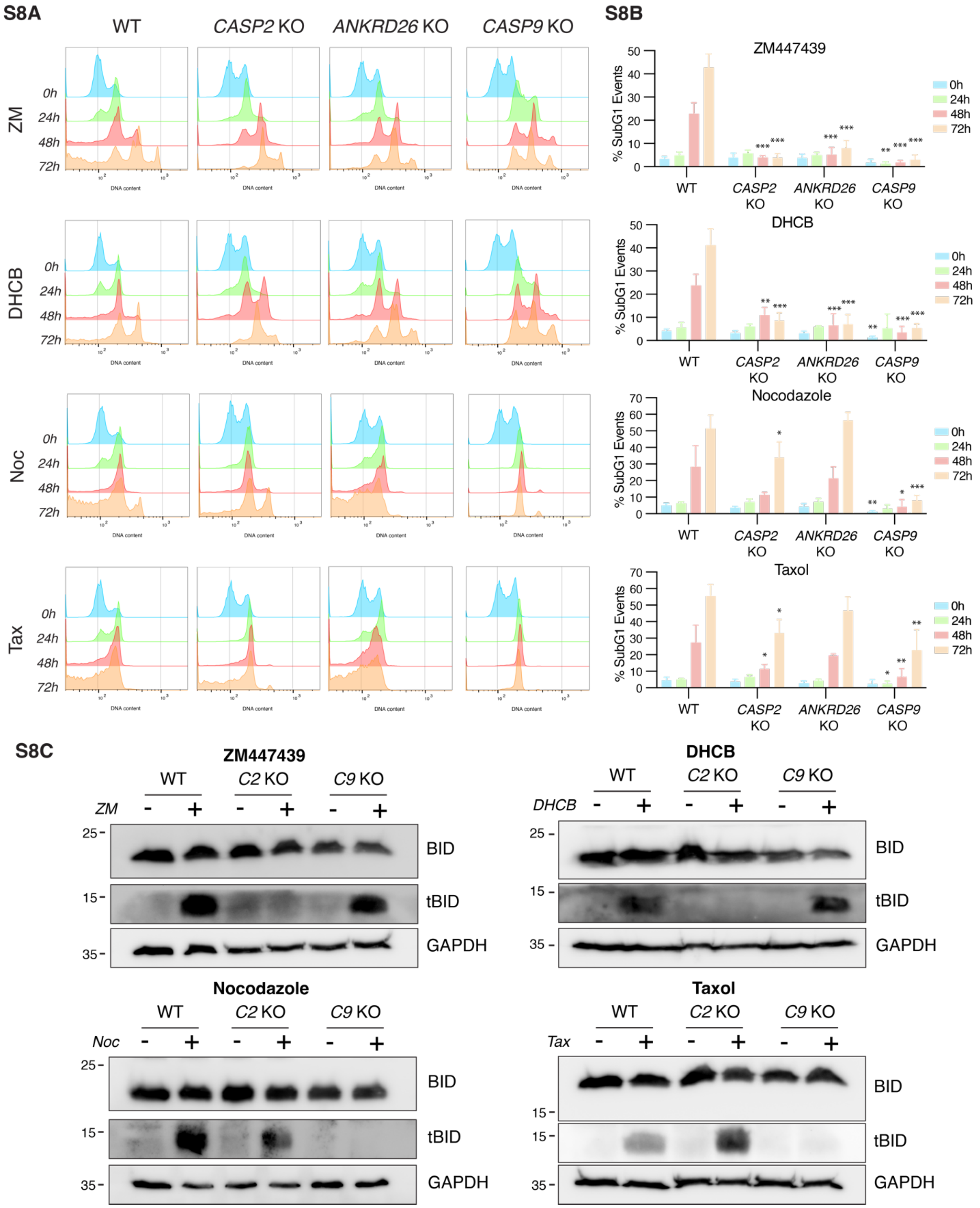

Fig. S8: Only cytokinesis failure elicits the centrosome-PIDDosome-tBID axis. (A) Representative DNA content profiles of Nalm6 cells WT and derivative clones lacking caspase-2, caspase-9 or ANKRD26 after different time points of exposure to 2µM ZM447439 (ZM), 4µM DHCB, 100nM Nocodazole (Noc) or 50nM Taxol (Tax). **(B)** Quantification of the percentage of subG1 events from B. Data is presented as means ± SD of N ≥ 3 independent biological replicates. Statistical significance was calculated by one-way ANOVA with Dunnett's multiple comparison test, comparing each

time point of the KO clone to the corresponding time point of the WT sample. * = p value < 0.05; ** = p value < 0.01; *** = p value < 0.001. **(C)** Representative western blots showing the processing of BID into tBID in Nalm6 cells WT or clones lacking caspase-2 or caspase-9 after 24h of treatment with the drugs described in A.

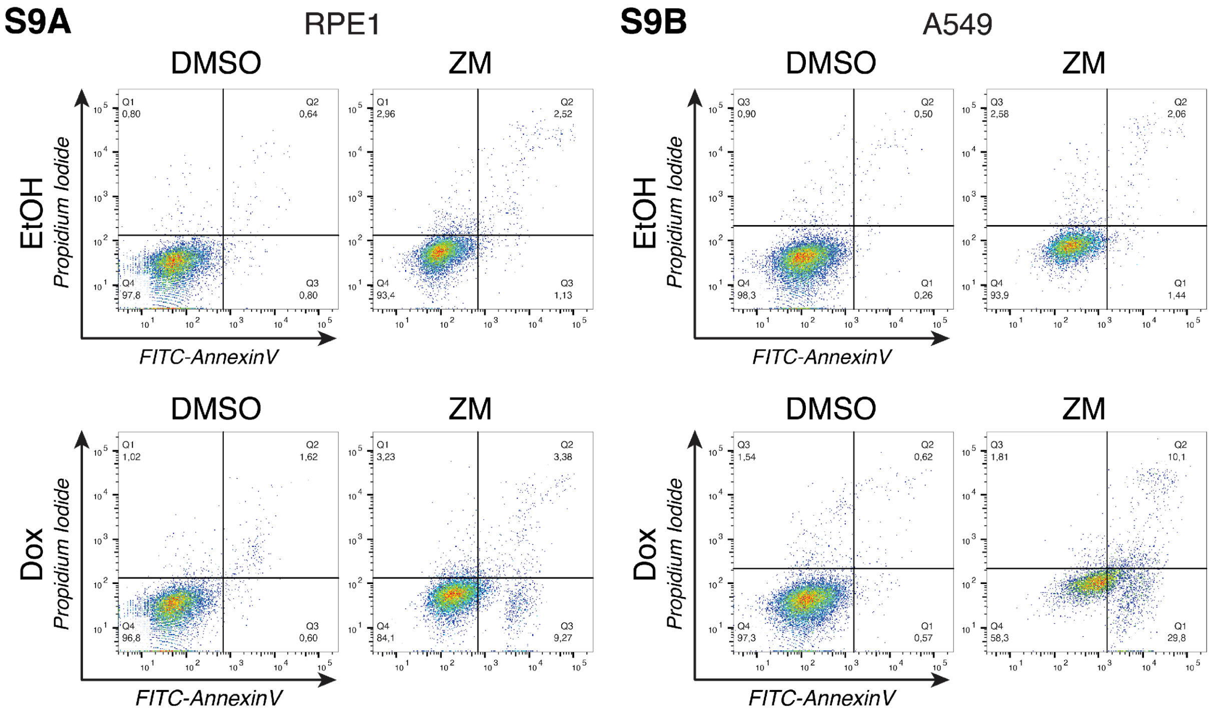

Fig. S9: BID overexpression sensitizes epithelial cells to cell death after cytokinesis failure. (A) Representative dot-plot examples of AnnexinV/PI staining followed by flow cytometric analysis of RPE1 cells overexpressing BID after treatment with 2µM ZM447439. **(B)** A549 cells treated the same as in A.

Table S1.
Results of the Taxol CRISPR Screen.

File “Table1 - Taxol gene selection.xlsx”

Table S2.
Results of the Taxol + Reversine CRISPR Screen.

File “Table2 - Tax+Rev gene selection.xlsx”

Table S3.
Supplementary Materials and Methods.

File “Table3 - Materials and Methods.xlsx”
